# Bayesian inference of frequency-specific functional connectivity in MEG imaging using a spectral graph model

**DOI:** 10.1162/imag_a_00307

**Published:** 2024-10-10

**Authors:** Huaqing Jin, Farras Abdelnour, Parul Verma, Benjamin S. Sipes, Srikantan S. Nagarajan, Ashish Raj

**Affiliations:** Department of Radiology and Biomedical Imaging, University of California San Francisco, San Francisco, CA, United States

**Keywords:** Bayesian, connectomes, functional connectivity, magnetoencephalography, spectral graph theory, simulation-based inference

## Abstract

Understanding the relationship between structural connectivity (SC) and functional connectivity (FC) of the human brain is an important goal of neuroscience. Highly detailed mathematical models of neural masses exist that can simulate the interactions between functional activity and structural wiring. These models are often complex and require intensive computation. Most importantly, they do not provide a direct or intuitive interpretation of this structure–function relationship. In this study, we employ the emerging concepts of spectral graph theory to obtain this mapping in terms of graph harmonics, which are eigenvectors of the structural graph’s Laplacian matrix. In order to imbue these harmonics with biophysical underpinnings, we leverage recent advances in parsimonious spectral graph modeling (SGM) of brain activity. Here, we show that such a model can indeed be cast in terms of graph harmonics, and can provide a closed-form prediction of FC in an arbitrary frequency band. The model requires only three global, spatially invariant parameters, yet is capable of generating rich FC patterns in different frequency bands. Only a few harmonics are sufficient to reproduce realistic FC patterns.

We applied the method to predict FC obtained from pairwise magnitude coherence of source-reconstructed resting-state magnetoencephalography (MEG) recordings of 36 healthy subjects. To enable efficient model inference, we adopted a deep neural network-based Bayesian procedure called simulation-based inference. Using this tool, we were able to speedily infer not only the single most likely model parameters, but also their full posterior distributions. We also implemented several other benchmark methods relating SC to FC, including graph diffusion and coupled neural mass models. The present method was shown to give the best performance overall. Notably, we discovered that a single biophysical parameterization is capable of fitting FCs from all relevant frequency bands simultaneously, an aspect that did not receive adequate attention in prior computational studies.

## 1 Introduction

A key goal in the neuroscience field is to understand the relationship between functional activity and the static anatomical structural wiring in the human brain (Fornito et al., 2015;Suárez et al., 2020). Many noninvasive neuroimaging techniques are used to measure functional activity, including functional magnetic resonance imaging (fMRI), electroencephalography (EEG), and magnetoencephalography (MEG) (da Silva, 2013;Tivadar & Murray, 2019). The most common way in which these data are utilized is in the form of a functional connectivity (FC) matrix, which specifies the correlation between any two brain regions’ measured activity time series (Bassett & Bullmore, 2009;Bullmore & Sporns, 2009). Although historically the field has focused on fMRI-derived FC, it is now also possible to employ MEG time series to obtain FC; for example,Brookes et al. (2011,^2012^) where the authors find spatial agreement between FC measured using MEG and fMRI. Structural wiring is typically obtained using diffusion tensor imaging (DTI) (Verma et al., 2022), followed by computational tractography to produce a matrix of structural connectivity (SC) that gives the connection strength between all pairs of gray matter regions (Bullmore & Sporns, 2009;Hagmann et al., 2008). Hence a powerful way to understand the structure–function relationship in the brain is by achieving a mapping between SC and FC.

Graph theory-based statistical relationships between SC and FC are well known phenomenologically (Abdelnour et al., 2014,^2018^;Achard et al., 2006;Bassett & Bullmore, 2006,^2009^;Buckner et al., 2005;Bullmore & Sporns, 2009;Chatterjee & Sinha, 2007;Ghosh et al., 2008,^2023^;He et al., 2008;Hermundstad et al., 2013;Mišić et al., 2014,^2016^;Park & Friston, 2013;Rubinov & Sporns, 2010;Rubinov et al., 2009;Strogatz, 2001;van den Heuvel et al., 2009;Xie et al., 2021). Most such SC-FC studies pertain to fMRI, but are increasingly being applied to M/EEG (Messaritaki et al., 2021;Wodeyar & Srinivasan, 2022). It has recently emerged that*graph harmonics*, also known as*eigenvectors*,*gradients*, or*eigenmodes*, of the SC matrix, especially its Laplacian, are excellent graph features for mapping this structure–function relationship. Indeed, the concept of graph harmonics, borrowing from the emerging field of graph signal processing (GSP) (Auffarth, 2007;Kondor & Lafferty, 2002;Larsen et al., 2006;Ng et al., 2001), provides an elegant and concrete mathematical framework to describe brain function and reflect distinct spatial patterns of functional signal (Abdelnour et al., 2014;Atasoy et al., 2016;Ghosh et al., 2023). It is of course not necessary to employ*graph*harmonics; it has been shown that eigenmodes of signal propagation operators in physical media (i.e., brain tissue), following its geometric boundaries, are also capable of producing closed-form solutions and in reproducing FC (Robinson et al., 2016). The geometric eigenmode approach (Pang et al., 2023) is utilized for both task-evoked and task-free fMRI data, suggesting that brain activity can be succinctly explained through the brain’s geometry. However, it remains to be investigated whether this conclusion also applies to MEG data. Despite these findings, graph theoretic methods may be preferable due to their simplicity and do not require detailed knowledge of an individual brain’s geometric conformations.

The graph harmonics of SC and FC appear to be shared, and their eigenvalues reflect their graph frequency (Chung, 1997;Deslauriers-Gauthier et al., 2020), and together they form a graph Fourier basis. Eigenvalues of FC are related by a function of SC eigenvalues (Becker et al., 2018;Deslauriers-Gauthier et al., 2020;Lioi et al., 2021;Meier et al., 2016;Robinson et al., 2016;Tewarie et al., 2020), for example, exponential (Abdelnour et al., 2021) or power series (Becker et al., 2018;Meier et al., 2016). The explanatory power of graph harmonics was further extended byXie et al. (2021)who, by introducing conductance delays between nodes, proposed a so-called complex Laplacian whose harmonics possessed rich spatial patterns that further improved the structure–function correspondence. Remarkably, only a few SC graph harmonics are usually sufficient to reproduce empirical fMRI-derived FC (Abdelnour et al., 2018;Atasoy et al., 2016;Preti & Van De Ville, 2019;Xie et al., 2021). Hence harmonics may be a natural organizing principle for structure–function mapping, whereby low harmonics are thought to couple to global or integrative functions, while high harmonics are uncoupled to function and subserve segregative functions (Preti & Van De Ville, 2019;Rué-Queralt et al., 2023). The SC’s Laplacian harmonics may be thus thought of as the essential substrate on which functional patterns of the brain are established (Abdelnour et al., 2018;Atasoy et al., 2016;Glomb et al., 2020;Preti & Van De Ville, 2019;Robinson et al., 2016;Xie et al., 2021).

Unfortunately the explanatory power of graph harmonics is limited to a phenomenological level, since they do not emerge from basic underlying biophysical processes, and are, therefore, unable to infer mechanistic insights (Mišić et al., 2015;Raj et al., 2022). Previously we and others showed that harmonics naturally arise from fMRI signal diffusion or random walk through the SC graph (Abdelnour et al., 2014;Deslauriers-Gauthier et al., 2020;Tewarie et al., 2020), but passive diffusion is too simple to describe the rich and oscillatory MEG signal. Historically, biophysical relevance necessitated detailed connectome-coupled neural mass models (NMM) whose parameters reflect actual biophysical processes such as excitatory and inhibitory neuronal gains and capacitances (Breakspear, 2017;Cabral et al., 2014,^2017^;David & Friston, 2003;Destexhe & Sejnowski, 2009;El Boustani & Destexhe, 2009;Honey et al., 2009;Muldoon et al., 2016;Siettos & Starke, 2016;Spiegler & Jirsa, 2013;Wilson & Cowan, 1973). Such approaches involve massive time consuming nonlinear simulations. Thus the role of SC in these models is observed only indirectly, and does not allow a harmonic decomposition of SC. Due to computational and interpretational challenges, NMMs are not well suited to reveal the key organizing role of graph harmonics in functional activity. Another conceptual limitation is that the mapping between SC and FC is done separately for each frequency band of interest, whether low-frequencydelta, dominantalpha,or high-frequencybeta. Model parameters that best fit one band typically do not fit another—this is problematic since the S–F coupling is a property of physical neural systems and should not be frequency band dependent. While it is possible that the coupling may exhibit an apparent frequency dependency, that dependency should come about from the model directly or from modeling assumptions (e.g., frequency-dependent noise or coherence properties).

In this study, we attempt to fill key conceptual and practical gaps in the field of brain graph harmonics and their relationship with FC obtained from MEG recordings. We begin by establishing the relationship between the eigenvalues of structural graph harmonics and those of FC. Then, we develop the theory necessary to explain how graph harmonics emerge from basic biophysical processes on SC, leveraging recent advances in modeling wide-band MEG power spectra using the spectral graph model (SGM) (Raj et al., 2020;Verma et al., 2022). Note, although the present harmonic model of FC retains the same biophysical principles as the SGM work, it is specifically designed to predict narrow-band FC, not wide-band power spectra. Despite the simplicity of the harmonic decomposition, we show that indeed the proposed theory is imbued with all detailed biophysical processes and parameters that have hitherto only been available via lengthy nonlinear NMM simulations. We show, for the first time, that MEG FC from multiple frequency bands can be*simultaneously*decomposed into a shared, parsimonious set (3–5) of low harmonics of SC. Remarkably, the proposed theory admits an analytical, closed-form solution of MEG FC—a rare feature in comparison with extant NMMs which are only revealed via large simulations. We then propose a speedy and flexible deep learning algorithm for inferring the biophysical parameters that dictate the SC–FC relationship based on this harmonic decomposition. This tool allows us to infer model parameters almost instantaneously for a given subject, after the network has been trained on sufficient simulation-based training samples. Parameter inference has historically been an intractable challenge in neural system modeling, and prior coupled NMMs require a combination of hand tuning and grid search (Glomb et al., 2022;Xie et al., 2019). In contrast, we were not only able to obtain best-fit biophysical parameters very quickly (within seconds) but also to give their full posterior distributions. The presented approach should also be contrasted with another popular FC inference method called dynamic causal modeling (DCM) (Kiebel et al., 2008;Pinotsis et al., 2012), which seeks to estimate effective connectivity from functional activity, and does not employ an explicit structure–function model as we do; see Discussion section for further details.

We demonstrate this inference ability, critical for potential practical applications of model-based SC–FC inference, on a study of 36 healthy individual subjects’ MEG recordings. We achieved excellent predictive power overdelta,theta,alpha, andbetafrequency bands, which favorably compared with several benchmark methods, including prior graph harmonic mapping and SC-coupled NMM methods. Remarkably, we find that a single biophysical parameterization is capable of simultaneously fitting FCs from all relevant frequency bands. Thus our third key contribution is to show that the same underlying biophysical model and the same graph harmonics can predict FC in all frequency regimes. By allowing for frequency dependency to emerge from the underlying biophysics of neural activity, we resolve one of the key conceptual limitations of NMMs—the biologically implausible inability of a single NMM model to fit to all FCs from all frequency bands simultaneously.

This theoretical and experimental study attempts to combine both the elegance of graph harmonics and the biophysical relevance of detailed NMMs. Thus the proposed method may be considered a “computational microscope” into which high-dimensional MEG data are funneled into produce low-dimensional inferred parameters that parsimoniously capture the SC–FC relationship. The latter may be further developed into subject-specific biomarkers of mental or disease state. The demonstration that parsimonious, biophysical harmonic-based SC–FC mapping can be achieved at near-instantaneous speed in individual subjects opens new scientific and practical avenues for the emerging science of brain harmonics.

## 2 Theory and Model

Our goal of achieving a closed-form solution of functional connectivity (FC) and relating it to structural connectivity necessitates a deterministic model of how neural activity, as measured by MEG recordings, becomes established on the brain’s structural connectome. For this purpose, we leverage the spectral graph model (SGM), first introduced inRaj et al. (2020)andVerma et al. (2022). Our modeling effort begins from the SGM due to several desirable properties. First, SGM is a linear biophysical model with a closed-form solution in the frequency domain. Consequently, compared with nonlinear neural mass models—where identifiability of model parameter is not guaranteed (Hartoyo et al., 2019;Raj et al., 2022;Xie et al., 2019), parameter inference with SGM is more tractable and faster. Prior analyses (Raj et al., 2020;Verma et al., 2022) show that SGM is able to recover the steady state wide-band frequency spectra as well as the spatial patterns of thealphafrequency band obtained from MEG. Finally, SGM is highly parsimonious, with only seven global biophysically interpretable parameters while other biophysical models typically require hundreds of spatially varying parameters.

We begin this section by first describing the SGM in brief; the reader is referred to the original publications for detailed derivations. It is emphasized that the prior SGM papers do not by themselves provide a recipe or closed-form solution of FC, which is the focus of the current study and will be described in detail in the subsequent section.

**Notation.**Scalar variables are denoted in normal font, while vectors and matrices are denoted in boldface. Matrices are typically uppercase while vectors are typically lowercase. Matrix complex conjugate, transpose, and Hermitian are denoted by${\left(\cdot \right)}^{*}$,${\left(\cdot \right)}^{T}$, and${\left(\cdot \right)}^{T}$, respectively. The expectation is denoted byε(⋅). Frequency is denoted by angular frequencyωin radians per second and is related to frequencyfin Hertz viaω=2πf.

### 2.1 Spectral graph modeling

Spectral graph model (SGM) is a hierarchical, linear, analytic model of brain oscillations, represented via eigendecomposition of the Laplacian of the structural connectivity matrix (Raj et al., 2020;Verma et al., 2022). In this sense, it is an exemplar of an emerging body of work on*harmonic models*of brain activity, whereby the latter is described as a finite superposition of the brain network’s harmonics or eigenvectors (Abdelnour et al., 2014,^2021^;Atasoy et al., 2016;Becker et al., 2018;Deslauriers-Gauthier et al., 2020;Meier et al., 2016;Tewarie, Abeysuriya, et al., 2019;Tewarie et al., 2022). In contrast to other harmonic-based models, the SGM provides an explicit wide-band frequency response, spanning low-frequency BOLD signal all the way to high-frequency MEG signal in the gamma band (Verma et al., 2022). A typical SGM has two model layers, a mesoscopic layer for local neural circuits in the cortex and a macroscopic layer for the whole brain, which accommodates the long-range fiber projections that connect individual local circuits and neural populations. The model, therefore, explicitly lays out how the structural connectome governs and mediates neural activity.

A salient feature of SGM is that it provides a closed-form solution of brain oscillations under the frequency domain. Notably, the model can be fully characterized by only seven global parameters$\theta ={\left(\tau_{\text{e}},\tau_{\text{i}},g_{\text{ei}},g_{\text{ii}},\tau_{\text{g}},v,\alpha \right)}^{\text{T}}$. These include excitatory and inhibitory time constants$\tau_{\text{e}}$,$\tau_{\text{i}}$, and neural gains$g_{\text{ei}}$and$g_{\text{ii}}$at the mesoscopic level; and long-range excitatory time constant$\tau_{\text{g}}$, speedv, coupling constantαat the macroscopic level. We define the complex Laplacian matrix$ℒ\left(\omega ;\alpha ,v\right)=\text{I}-\alpha {\text{C}}^{*}\left(\omega ;v\right),$where the elements of${\text{C}}^{*}$are$c_{ij}^{*}\left(\omega ;v\right)=c_{ij}\text{exp}\left(-\text{j}\omega \tau_{ij}^{v}\right)$,i,j=1...N, where$c_{ij}$is the structural connectivity between regionsiandjand$\tau_{ij}^{v}$corresponds to the delay due to a finite speedv, which is calculated for every region as the distance between regions divided by the speedv. As derived inRaj et al. (2020)andVerma et al. (2022), the frequency domain solution of the macroscopic signals is given in closed form as

$$\text{X}(\omega )={\left(\text{j}\omega \text{I}+\frac{1}{\tau_{\text{g}}}F_{\text{g}}(\omega )ℒ(\omega )\right)}^{-1}H_{\text{local}}(\omega )\text{P}(\omega ),$$ (1)

where$F_{\text{g}}(\omega )$is the Fourier transform of a gamma-shaped neural response function$f_{\text{g}}\left(t;\tau_{\text{g}}\right)$that seeks to model the macroscopic transfer function of excitatory projection neurons, with characteristic time constant$\tau_{\text{g}}$. The function$H_{\text{local}}\left(\omega ;\tau_{\text{e}},\tau_{\text{i}},g_{\text{ei}},g_{\text{ii}}\right)$represents the transfer function capturing the overall activity of populations of excitatory and inhibitory neurons at the local or mesoscopic level. SGM allows for fitting local transfer function to each region; however, it was previously shown that this is not necessary and that a spatially invariant set of mesoscopic parameters is sufficient to recapitulate empirical MEG power spectra (Raj et al., 2020). The entire model is driven by external or region-specific processp(t), whose Fourier transform is denotedP(ω)above.

Factoringℒ(ω)into its eigen components$ℒ(\omega )=\text{U}(\omega )\Lambda (\omega ){\text{U}}^{H}(\omega )=\sum_{k=1}^{N}\lambda_{k}(\omega ){\text{u}}_{k}(\omega ){\text{u}}_{k}^{H}(\omega ),$we can writeEquation (1)as an expansion on the harmonics ofℒ:

$$\text{X}(\omega )=\sum_{k=1}^{N}\frac{{\text{u}}_{k}(\omega ){\text{u}}_{k}^{H}(\omega )}{\text{j}\omega +\tau_{\text{g}}^{-1}\lambda_{k}(\omega )F_{\text{g}}(\omega )}H_{\text{local}}(\omega )\text{P}(\omega ).$$ (2)

For reference, a detailed derivation is given in the accompanyingSupplementary Informationsection.

### 2.2 Deriving a closed-form expression for cross-spectral density of SGM

Prior use of SGM has focused on obtaining the neural signal equation in the frequency domain and determining its power spectrum. Here we propose to explicitly derive the*structure–function relationship*, that is, the second order statistics of the signalXin terms of the eigendecomposition of the structural Laplacianℒ. There are several equivalent ways to achieve this; here we use the most intuitive approach, starting with a definition of the cross-spectral density (CSD) as the expectation$\varepsilon \left(X(\omega )X^{H}(\omega )\right)$. Let us rewriteEquation (2)more succinctly as$\text{X}(\omega )=\text{U}(\omega )\Gamma (\omega ){\text{U}}^{H}(\omega )H_{\text{local}}(\omega )\text{P}(\omega )$, where the diagonal matrixΓ(ω)has as its k-th diagonal entry$\gamma_{k}(\omega )=\frac{1}{\text{j}\omega +\tau_{\text{g}}^{-1}\lambda_{k}(\omega )F_{\text{g}}(\omega )}$. Note that the model is dependent on a given instantiation of the model parametersθ; for convenience, this dependence is not shown above but is always to be assumed implicitly. Using this shorthand, we expand the CSD ofX(ω)as

ε(X(ω)XH(ω))= |Hlocal(ω)|2U(ω)Γ(ω)UH(ω)ε(P(ω)PH(ω))U(ω)ΓH(ω)UH(ω).

Now, we introduce the key assumption that*the driving input signal is uncorrelated across regions and across time*. This is plausible because we are interested in achieving the resting-state stationary description of FC, where no external stimulation is provided to the brain. In this scenario, the driving signal is internal to the brain, and while those internal processes may have spatial heterogeneity at specific instances, over time those reflect simply as spatially and temporally uncorrelated noise processes. With this assumption or approximation, we have$\varepsilon \left(\text{P}(\omega )\;{\text{P}}^{H}(\omega )\right)=\sigma^{2}\text{I}$for an i.i.d. noise process with variance$\sigma^{2}$. Since the harmonics or eigenvectors are orthonormal, we have${\text{U}}^{H}(\omega )\;\text{U}(\omega )=I$, and the right-hand side of the above equation greatly simplifies to$\sigma^{2}{\left|H_{\text{local}}(\omega )\right|}^{2}\text{U}(\omega ){\left|\Gamma (\omega )\right|}^{2}{\text{U}}^{H}(\omega )$.

Additionally, the estimated FC is subsequently normalized via its diagonal terms, which eliminates the variance$\sigma^{2}$and local frequency response function$H_{local}(\omega )$, which no longer needs to be inferred.

Thus, finally, we have a closed-form solution of the CSD$\hat{F}(\omega )$at any frequency, as a superposition over graph harmonics:

$$\hat{\text{F}}\left(\omega ,\theta_{reduced}\right)=\sum_{k=1}^{N}\frac{{\text{u}}_{k}(\omega ){\text{u}}_{k}^{H}(\omega )}{{\left|\text{j}\omega +\tau_{\text{g}}^{-1}\lambda_{k}(\omega )F_{\text{g}}(\omega )\right|}^{2}}.$$ (3)

Note, the theoretical FC is now dependent on a reduced set of model parameters, since due to the elimination of$H_{local}(\omega )$term, the estimated FC from SGM only depends on the three-parameter reduced vector$\theta_{reduced}=\left(\tau_{\text{g}},v,\alpha \right)$.

Thus, the theoretical FC of the SGM is given by a superposition of graph Laplacian eigenmodes or harmonics$u_{k}(\omega )$, each of which manifests a frequency response$\gamma_{k}(\omega )$dependent on the eigenvalue$\lambda_{k}(\omega )$. The eigenvectors of the predicted FC are identical to those of the structural Laplacian. In this manner, we have reduced the full cross-spectral density of brain activity to modeling just the diagonal eigenvalues of the structural connectome; all region-pair coherences are thus expected to be captured entirely by the eigenvectorsU(ω). This description is entirely consistent with prior studies using graph harmonics (Abdelnour et al., 2014,^2021^;Atasoy et al., 2016;Becker et al., 2018;Deslauriers-Gauthier et al., 2020;Meier et al., 2016;Tewarie, Abeysuriya, et al., 2019;Tewarie et al., 2022), but in contrast to those studies, here we provide for the first time a fully frequency-resolved description of FC, at arbitrary frequency. Remarkably, this description is direct, and does not require either simulations in time, or indirect calculation of FC via the narrow-band Hilbert envelope (Cabral et al., 2014;Tewarie et al., 2022).

## 3 Methods

### 3.1 Dataset

In this work, we analyze magnetoencephalography (MEG) data. The dataset is based on preprocessed and publicly available dataset for the SGM work (Xie et al., 2019), and is identical to the one used for the modified SGM (Verma et al., 2022). The MEG datasets and the corresponding SC and distance matrices were all processed at the regional level under the Desikan–Killiany atlas parcellation with68cortical regions (Desikan et al., 2006). For this dataset, MEG, anatomical MRI, and diffusion MRI were collected for36healthy adult subjects (23males,13females;26left handed,10right handed; mean age21.75years, with age range7–51years). Data collection procedure has been described previously (Raj et al., 2020). All study procedures were approved by the institutional review board at the University of California at San Francisco and were in accordance with the ethics standards of the Helsinki Declaration of 1975 as revised in 2008. MEG recordings were collected for5minutes while the subjects were resting and had eyes closed. Out of the5-minute recording, a1-minute snippet was chosen which was deemed most noise free. MRI followed by tractography was used to generate the connectivity and distance matrices. The entry in the distance matrix is the fiber length distance, which is defined as the average number of voxels spanned across all streamlines between those regions. The publicly available dataset consisted of processed connectivity and distance matrices for every subject.

For details on the data processing, refer to Section S.1 of theSupplementary Materialsas well as the literature (Jin et al., 2023;Raj et al., 2020;Verma et al., 2022).

### 3.2 Constructing functional connectivity (FC) in discrete frequency bands

This study requires the fitting of an FC matrix derived from the theoretical cross-spectral density (Eq. 3) to empirical MEG-derived FC, specifically its inter-regional coherence. We, therefore, evaluated FC at four commonly studied MEG frequency bands:delta(2–3.5Hz),theta(4–7Hz),alpha(8–13Hz), andbeta(13–20Hz).

Since the SGM was tailored to frequencies up to≈25Hz, we only investigate the results on the lower part of thebetaband (i.e.,13–20Hz). The SGM is designed for frequencies up to around 25 Hz because our SGM can accurately reconstruct the PSD below25Hz, that is, the beta band. Higher frequencies, in the gamma band, arise mainly from local neural populations that do not appear to be modulated by the structural connectivity, hence a connectome-based model such as SGM would not be expected to fit spectra in this regime, as noted in prior reports (Jin et al., 2023;Raj et al., 2020;Verma et al., 2022).

In order to convert the theoretical CSD to band-specific FC, we first summed the CSD across all frequencies within a given band, then normalized its rows and columns by the diagonal. For example, for thealpha-band we define

$$\begin{array}{ccc} {\hat{\text{F}}}_{alpha}\left(\theta_{reduced}\right)=\Delta^{-1/2}\int_{\omega \in \Omega_{alpha}}\hat{\text{F}}\left(\omega ,\theta_{reduced}\right)d\omega \Delta^{-\frac{1}{2}}, & & \end{array}$$ (4)

whereΔcontains the diagonal entries of the band sum$\int \hat{\text{F}}(\omega )d\omega$and$\Omega_{alpha}$contains the range of frequencies within thealphaband. Analogously, we define${\hat{\text{F}}}_{delta,}$${\hat{\text{F}}}_{theta}$and${\hat{\text{F}}}_{beta}$; generically, we will refer to a given band’s FC as${\hat{\text{F}}}_{band.}$

Empirical FC matrices using MEG data were constructed analogously to the theoretical ones, using coherence-based analysis implemented in the MNE-Connectivity 0.4.0 Python toolbox (Gramfort et al., 2013). Denoting the Fourier transform of two time series forith andjth ROIs as$Y_{i}(\omega )$and$Y_{j}(\omega )$, thei,j-th entry of coherence-based FC for a given frequency band, sayalpha, is given by

$$\begin{array}{ccc} {\left[{\text{F}}_{alpha}\right]}_{i,j}=\frac{\left|\int_{\omega \in \;\Omega_{alpha}}Y_{i}^{*}(\omega )Y_{j}(\omega )d\omega \right|}{\left|\int_{\omega \in \;\Omega_{alpha}}Y_{i}(\omega )d\omega \right|\;\left|\int_{\omega \in \;\Omega_{alpha}}Y_{j}(\omega )d\omega \right|}. & & \end{array}$$ (5)

It may be verified that both the theoretical and empirical FCs are analogous to coherence, and normalized in the same manner. In this work, we set the diagonal elements of both theoretical and empirical FC to zero following the convention. Our implementation replaces the integration above by summation over 10 equally spaced frequency points per band. We used the spectral_connectivity_epochs function from the MNE-Connectivity Python package (Gramfort et al., 2013), set to 100 epochs, to obtain the FCs from the MEG recordings for each frequency band. Specifically, this function firstly filters the MEG time series into the desired frequency band, then computes the coherence matrix for each epoch, and finally averages the matrices across epochs to obtain the FC matrix. Epochs are equal-duration time segments of the EEG/MEG signal during each of which the signal may be considered relatively stationary (Gramfort et al., 2013). Thus, the use of a single epoch reflects that the FC networks were obtained from the entire temporal signal, while>1epochs involve segmenting the time series and combining each segment’s FC together in a principled manner. The mode was set to multitaper, an option that has favorable time–frequency product and a optimal trade-off between bias and variance of spectral estimates. No frequency windowing was applied (mt_windowing).

### 3.3 Simulation-based inference for FC

Despite the parsimony and explicit solution of the SGM, its inference from empirical FC is quite challenging using sampling or gradient descent methods. Simulation-based inference (SBI) (Tejero-Cantero et al., 2020) is a recent advanced neural network-based tool that makes Bayesian inference possible for models with complex and stochastic simulators. This technique is especially attractive in situations where the likelihood function is intractable or difficult to compute, where other methods based on explicit formulation of the likelihood function become challenging. It shows some success in identifying mechanistic models of neural dynamics (Gonçalves et al., 2020) and especially presents promising results for predicting power spectral density with SGM for MEG data (Jin et al., 2023).

Here we adapt and apply the SBI tool to fit the SGM to empirical FC from selected frequency bands of MEG recordings (referred to as SGM-SBI). SBI requires the forward computation of a large number of simulations, in order to assemble a set of paired samples for training. Therefore, we first compute, for any parameter choice$\theta_{reduced}$drawn from a suitably defined prior distribution, the pair$\left(\theta_{reduced,}{\hat{\text{F}}}_{band}\left(\theta_{reduced}\right)\right)$for each frequency band,band∈{delta, theta, alpha, beta}, using the SGMEquations (3)and(4). Subsequently, noise from a standard Gaussian distribution is added to each entry of the computed mean FC matrix to account for the inherent noise in the empirical FC (Jin et al., 2023).

#### 3.3.1 Parameter transformation

Any biophysical model with physically realizable parameters typically admits well-defined parameter ranges beyond which the parameters and the overall model become implausible. The SGM too is a biophysical model whose parameters have well-defined biological meaning, and whose ranges can be specified*a priori*—seeTable 1. These ranges are taken from previous SGM works (Jin et al., 2023;Raj et al., 2020;Verma et al., 2022). The bounded parameter constraints cause difficulties for posterior sampling with SBI (Deistler et al., 2022), we reparameterize SGM parameters to ease the posterior sampling. Specifically, SGM parameters are transformed with a standard logit function$\text{s}=ℋ\left(\theta_{reduced}\right)$so that the original lower and upper bounds map to−∞and+∞, respectively (Jin et al., 2023).

**Table 1.** Global SGM parameters and bounds for parameter estimation for SBI-SGM.

Name	Symbol	Lower/upper bound
Graph time constant	$\tau_{\text{g}}$	[0.005 s, 0.03 s]
Transmission speed	v	[5.0 m/s, 20.0 m/s]
Long-range connectivity coupling constant	α	[0.1, 1]

#### 3.3.2 Prior specification

To obtain the satisfactory inference performance with SBI, a large training sample size is typically required. To ease the computational burden, we construct informative priors derived from the dual annealing fitting results to improve the quality of the simulated samples in the current study. With better quality samples, the simulated sample size can be significantly reduced and the neural network can learn the posterior distribution more effectively. To be specific, we firstly fit the theoretical and empirical FCs with the dual annealing algorithm with200iterations yielding the rough point estimate of SGM parameters,${\hat{S}}_{\text{ANN}}$. We adopt a Gaussian prior$\pi (\text{s})∼N\left({\hat{\text{s}}}_{\text{ANN}},\;\text{I}\right)$for the transformed parameters, where**I**represents the identity matrix.

#### 3.3.3 Training procedure

We adopt a three-round training process. Initially, 1000 pairs of$\left(\text{s},\;{\hat{\text{F}}}_{band}\left({ℋ}^{-1}(\text{s})\right)\right)$are generated using SGM and priorπ(s). These pairs are then used to train a neural network, parametrized byΦ, whose task is to approximate the true posterior ofsvia a neural spline flow$q_{\Phi }$(Durkan et al., 2019). Neural network architecture including50hidden features uses the two-block residual net for context embedding, which is the default setting in the SBI package (Tejero-Cantero et al., 2020). All other hyperparameters also adopt the default settings. We feed the empirical FC${\text{F}}_{band}$to$q_{\Phi }$, yielding the posterior distribution$q_{\Phi }\left(\text{s}|{\text{F}}_{band}\right)$with estimated parameter$\hat{\Phi }$for the first round. In the subsequent training round, we update parameters$\hat{\Phi }$in a similar manner. The only variation is that we use the previous round’s posterior distribution as the current round’s prior distribution for generating simulation pairs. After three-round training, the final estimation of the posterior$q_{\Phi }\left(\text{s}|{\text{F}}_{band}\right)$is obtained. The target posterior distribution of$\theta_{reduced}$is$q_{\hat{\Phi }}\times |\det(\text{J})|,$whereJis the Jacobian matrix of the transformation functionℋ(Henderson & Searle, 1979). The process of obtaining the posterior distribution ofsis delineated inAlgorithm 1. A more comprehensive illustration of the combination of SBI with SGM can be found inJin et al. (2023).

**Table:** 

**Algorithm 1.** Posterior estimation from FC with SBI
**Require:** An initial multivariate Gaussian prior $\pi (s)∼N({\hat{s}}_{ANN},I)$ , a specific frequency band band∈{delta, theta, alpha, beta} , SGM ${\hat{\text{F}}}_{band}\left({ℋ}^{-1}(\text{s})\right)$ , an observation ${\text{F}}_{band}$ , the number of samples per round M=1000 . **for** r=1,2,3 **do** **for** m=1, ... ,M **do** Sample ${\text{s}}_{m}∼\pi (\text{s})$ Compute SGM forward model ${\hat{\text{F}}}_{band}^{\left(m\right)}\left({ℋ}^{-1}({\text{s}}_{m})\right)$ **end for** $\hat{\Phi }\leftarrow \underset{\Phi }{\text{argmin}}-\frac{1}{M}\sum_{m=1}^{M}\text{log}\left\{q_{\Phi }\left({\text{s}}_{m}|{\hat{\text{F}}}_{band}^{\left(m\right)}\right)\right\}$ $\pi (\text{s})\leftarrow q_{\hat{\Phi }}\left(\text{s}|{\text{F}}_{band}\right)$ **end for** ** return $q_{\hat{\Phi }}\left(\text{s}|{\text{F}}_{band}\right)$ ** as the estimate of the posterior distribution of s . The posterior distribution of $\theta_{reduced}$ is $q_{\hat{\Phi }}\times \;\left|\text{det}(\text{J})\right|$ , where J is the Jacobian matrix of the transformation ℋ .

We also consider training jointly with all four bands, where the corresponding FCs are simply stacked into a larger matrix${\text{F}}_{shared}={\left({\text{F}}_{delta}^{T}{\text{F}}_{theta}^{T}{\text{F}}_{alpha}^{T}{\text{F}}_{beta}^{T}\right)}^{T}$, and analogously we define the larger theoretical FC matrix${\text{F}}_{shared}\left(\theta_{reduced}\right)$. Then the above algorithm is applied to these larger “all-bands” FCs, following which we infer the posterior of a single set of parameters$\theta_{shared}$that can simultaneously fit to all bands (referred to as SGM-SBI-shared).

### 3.4 Metrics of performance

To better evaluate the performance of our SGM-SBI pipeline in FC, we introduce three main metrics to assess the similarity between estimated and empirical FCs, including the Pearson’s correlation, Lin’s correlation (Lin, 1989), and mean squared error (MSE). Lin’s correlation (or concordance correlation coefficient) is a reproducibility index which has been used in the neuroimaging area (Lange et al., 1999) for a long time. While Pearson’s correlation only cares about the dependence between two variables, it further considers the deviation of the means and variances of the two variables. Thus, it can be regarded as a stricter version of Pearson’s correlation. The value of Lin’s correlation is within[−1,1],where a larger value indicates higher consistency.

While the interpretation of the values of Pearson’s correlation is straightforward, the values of Lin’s correlation and MSE lack intuitive understanding. Therefore, instead of the raw Lin’s correlation and MSE, we report the corresponding standardized versions. To be specific, we randomly shuffle the empirical FC for1000times and calculate the metrics between the shuffled versions and original one. Lin’s correlation and MSE are then standardized using the mean and variance derived from the shuffled metrics.

### 3.5 Comparison with benchmark methods

In order to compare the present SGM-SBI method for mapping FC from SC, we implemented several current and relevant benchmark methods. All benchmarks, described below, were applied to the same datasets as the proposed method, and were evaluated using the same performance metrics. The benchmark methods were:

1. *Direct mapping*. We used the subject’s SC itself as a direct prediction of their FC, measuring its performance using measures of correlation, Lin concordance, and MSE applied element-wise between the two matrices SC and FC.
2. *Eigen-mapping*. Recent work (Abdelnour et al., 2014,^2021^) shows that FC is predictable from the eigendecomposition of SC via a simple graph diffusion model. In the harmonics space, this amounts to a simple relationship between the eigenvalues in FC and SC Laplacian, for example, the exponential decay function. Hence we employed the two-parameter exponential relationship suggested byAbdelnour et al. (2021), that is,
  $$\begin{array}{cccc} \lambda_{\text{eig}}=\text{exp (}-\lambda_{\text{sc}}*p_{1}\text{)}+p_{2}, & & & \end{array}$$
  where$\left(p_{1},p_{2}\right)$are the parameters that need fitting and$\lambda_{\text{eig}}$and$\lambda_{\text{sc}}$are the eigenvalues of estimated FC from the eigen-mapping and SC, respectively.
3. *Coupled NMM*. We adopt the Wilson–Cowan (WC) model (Wilson & Cowan, 1972) as our NMM. To model the coupling between different brain regions, the WC model includes the input from other ROIs during its evolution. Specifically, denotingE(i,t)andI(i,t)as the number of excitatory and inhibitory cells firing at ROIiand timet, the model is

$$\begin{array}{l} \tau_{e}\frac{E\left(i,t\right)}{\text{d}t}=-E\left(i,t\right)+\left\{1-E\left(i,t\right)\right\}S_{e}\left\{c_{ee}E\left(i,t\right)-c_{ie}I\left(i,t\right)+O\left(i,t\right)+P\left(i,t\right)\right\}+\epsilon_{e}\left(i\right),\\ \tau_{i}\frac{I\left(i,t\right)}{\text{d}t}\;=-I\left(i,t\right)+\left\{1-I\left(i,t\right)\right\}S_{i}\left\{c_{ei}E\left(i,t\right)-c_{ii}I\left(i,t\right)+Q\left(i,t\right)\right\}+\epsilon_{i}\left(i\right), \end{array}$$

whereO(i,t)is the input from other ROIs,$\tau_{e}$and$\tau_{i}$are the time constants,$S_{e}$and$S_{i}$are functions of sigmoid form,$\left(c_{ee},c_{ie},c_{ei},c_{ii}\right)$are local coupling parameters,P(i,t)andQ(i,t)are the external input to the excitatory/inhibitory populations, and$\epsilon_{e}\left(i\right)$and$\epsilon_{i}\left(i\right)$are the noise. We implement the NMM with neurolib package in python (Cakan et al., 2021). NMM is fitted with the evolutionary algorithm over four parameters, global coupling strength, the baseline external input parameters (used to generateP(i,t)andQ(i,t)), and the global noise intensity. The simulation duration is set to61,000ms. For more detailed information on the implementation of the WC model, refer toCakan et al. (2021).

It is worth noting that the number of parameters in SGM-SBI, NMM, and eigen-mapping method are 3, 4, and 2, respectively, which are overall comparable. All benchmark methods above were fitted to and evaluated with Lin’s correlation loss. For the fitting process, we convert the FC matrix into a vector and apply the minmax operator on this vector for both SBI-SGM and NMM. However, in the case of the eigen-mapping method, we refrain from using the minmax operator as it prevents convergence. All performance metrics are computed with the vectorized FC following minmax operator. For all the methods, the SCs are preprocessed in identical ways, including removing the extreme connections via capping large values and adding minor values to enhance the connection between the corresponding regions between left and right hemispheres. These preprocessing steps are consistent with the previous work (Raj et al., 2020;Verma et al., 2022) and are robust to different datasets and choice of atlas.

## 4 Results

### 4.1 SGM produces rich FC patterns depending on parameters and frequency bands

We first need to establish that the proposed SGM is capable of producing a rich diversity of FCs that can change depending on frequency band and model parameters. We varied a single SGM parameter, in turn, in the range given inTable 1while fixing the other two at the optimal values fitted on empirical data. The predicted FCs are shown inFigure 1. All three SGM parameters influence the resulting FCs. However, their effects can vary significantly across different frequency bands. Indeltaandthetabands, each parameter noticeably affects overall connectivity strength as well as inter- and intrahemispheric connectivity. Model FCs generally exhibit denser and stronger connections within hemisphere and weaker connections between hemispheres. Inalphaband,$\tau_{G}$andαexert substantial influence within each hemisphere, while speedvpredominantly affects interhemispheric connectivity. Inbetaband,$\tau_{G}$andαshow minor effect on the FCs while increasingvreduces overall connectivity. Overall, the highest predicted FC is seen in lower frequencies, whilebetaband gives the lowest FC. Most importantly,*the same model, with fixed parameters, is capable of producing diverse FC patterns in different frequency bands*. The requirement that the same biophysical process be capable of producing frequency-specific FC was one of the key motivations of this study.

**Fig. 1.**
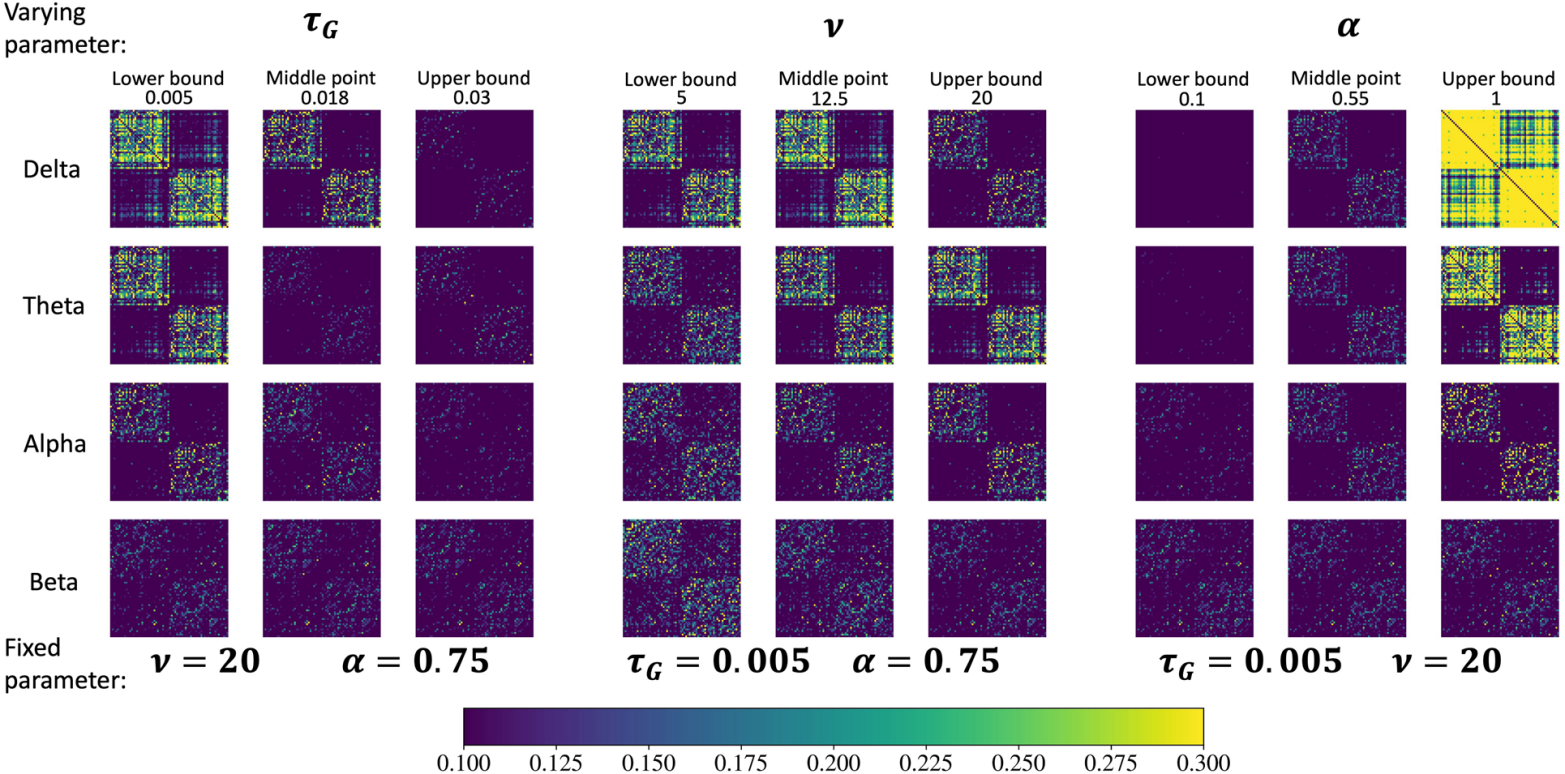
The SGM-modeled FCs indelta,theta,alpha, andbetabands when varying$\tau_{G}$(left),v(middle), andα(right) and fixing the other two parameters, separately. Note that each column of FCs shares the same SGM parameters. The fixed parameters are set to the fitted value from SGM-SBI-shared, while the varying parameters fluctuate within the range specified inTable 1. The values of three parameters are shown in the figure.

### 4.2 SC harmonics encompass spatial gradients of MEG FC

Since the proposed SGM is fundamentally based on the utility of SC harmonics to capture functional activity patterns, we first show inFigure 2Athe real components of the first three harmonics**u_1_**to**u_3_**on the brain surface. These harmonics, averaged across 36 subjects, are normalized within a range of[−1,1]. The first harmonic represents a global spatial pattern, previously suggested to represent the global signal in fMRI (Abdelnour et al., 2021). The second harmonic manifests a strong left–right spatial gradient and appears to capture the prominent interhemispheric functional connectivity structure. It also has a prominent internal structure with high levels in temporal and orbitofrontal areas and lower levels in medial and motor areas. Given that the brain plots exhibit similarity across various frequency bands, we have chosen to present plots from only one band. The third harmonic is laterally symmetric and captures a strong anterior–posterior gradient such that visual areas are at one pole and dorsolateral and orbitofrontal areas at the opposite pole. This finding is consistent with previous studies (Atasoy et al., 2016;Xie et al., 2021).

**Fig. 2.**
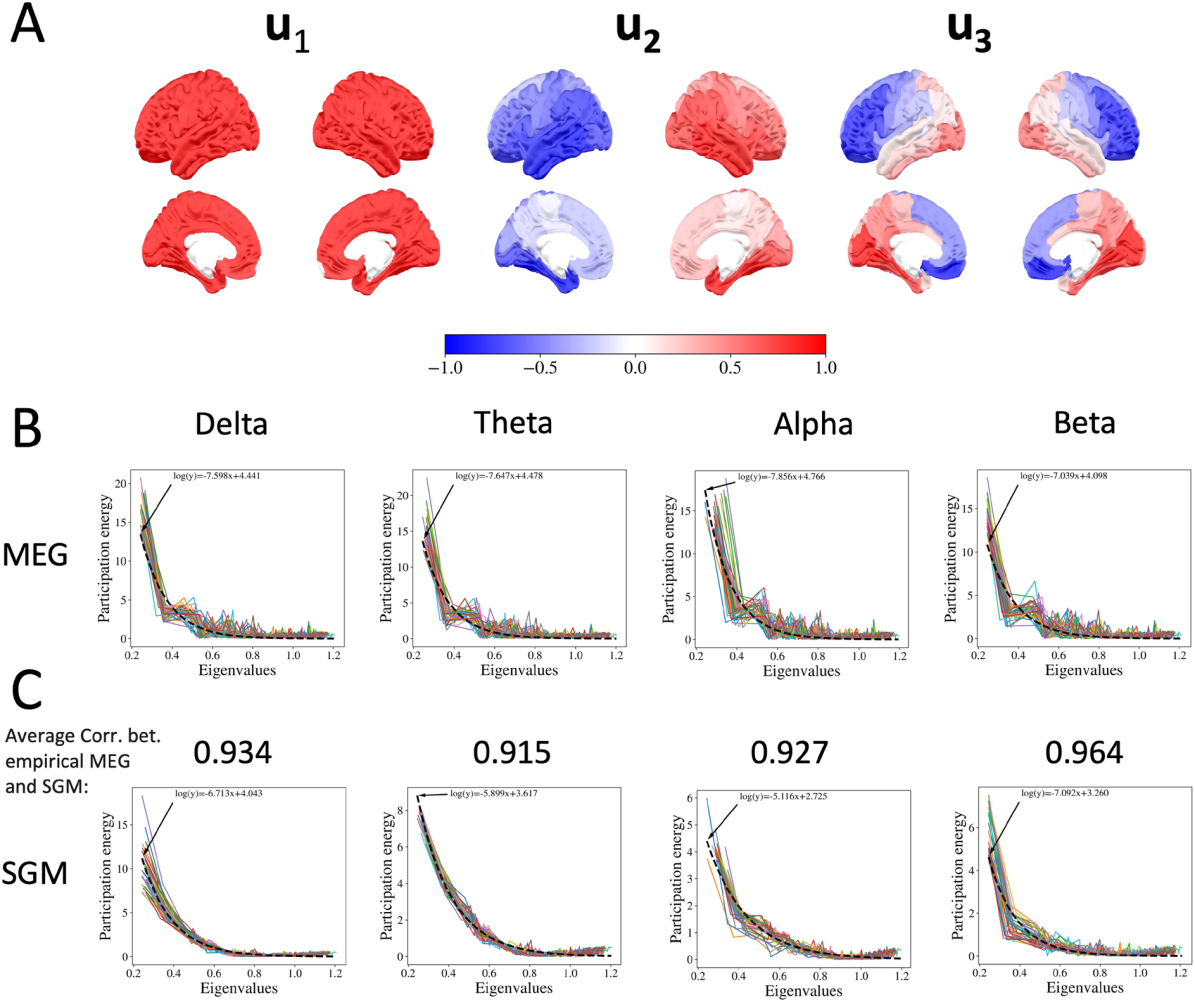
(A) The real part of first 3 complex Laplacian eigenvectors at thealphaband averaged on 36 subjects plotted on the brain. (B) The participation energy of empirical FCs after projecting them on the complex Laplacian eigenvectors versus the moduli of Laplacian eigenvalues fromdelta,theta,alpha, andbetabands. The black dashed lines represent the exponential decay function fitted to the participation energy of empirical FCs. (C) The participation energy of SGM-estimated FCs after projecting them on the complex Laplacian eigenvectors versus the moduli of Laplacian eigenvalues fromdelta,theta,alpha, andbetabands (same order as penal B). The black dashed lines represent the exponential decay function fitted to the participation energy of empirical FCs. The number above each plot is the average Pearson’s correlation between participation energies from MEG and SGM across36subjects. Since SGM is capable of giving wide-band spectra, each harmonic can have a different participation energy at different frequencies. Hence we show each frequency band’s participation energy in separate plots. The participation energy is defined as the moduli of diagonal elements of the projections of the FCs on the complex Laplacian harmonics, that is,$\text{diag}\left(U^{H}FU\right)$. Each curve represents a single subject. The empirical and theoretical participation energy curves show striking similarities with exceptionally high Pearson’s correlations (above 0.9 for all four bands).

### 4.3 Top few SC harmonics capture most of the energy of MEG FC

We evaluated the projections of the MEG-derived FC matrix$F_{band}$on the complex Laplacian harmonicsU, that is,$\text{diag}\left(U^{H}F_{band}U\right)$. The moduli of diagonal elements of this matrix contain the amount of MEG FC that is captured within each harmonic. Let us refer to this quantity as a harmonic’s “participation energy.” The relationship between the SC harmonics’ participation energy in MEG FC and the moduli of their associated eigenvalues is shown inFigure 2B, separately for each frequency banddelta,theta,alpha, andbeta. Each curve corresponds to a single subject. In producing these plots, the Laplacian matrix was evaluated at each band’s mean frequency, for example, frequencies[2.75,5.5,10.0,16.5]Hz. Across all bands, the MEG FC appears to be well captured by only the first few harmonics, roughly**u_1_**to**u_3_**. It is closely related to the observation inTokariev et al. (2019), where the authors showed that low order brain surface eigenmodes carry most of the energy. Interestingly, the eigenvalue relationship appears to follow roughly the exponential decay function proposed by our group on fMRI data based on graph diffusion arguments (Abdelnour et al., 2014). This exponential relationship is well established now for fMRI FC but has not previously been demonstrated for MEG data. Nonetheless, there are many deviations from a strict exponential curve, especially in the second and third harmonics’ participation energy.

### 4.4 SGM recapitulates the eigenvalue relationship between SC and MEG FC

Next we assessed whether the theoretical SGM manifests a similar harmonic decomposition. To do this, we obtained the participation energies of all harmonics for the model-predicted FC${\hat{F}}_{band}$given by$\text{diag}\left(U^{H}{\hat{F}}_{band}U\right)$. We chose the mean posterior SGM parameters from the band-specific SGM-SBI to produce the predicted FC in each band; detailed results of model fitting are contained in later sections. The resulting plots across all bands is shown inFigure 2Cwhere the Pearson’s correlation between participation energies from MEG and SGM is shown above each plot. The empirical and theoretical participation energy curves show striking similarities with exceptionally high Pearson’s correlations (above 0.9 for all four bands), and a very similar relationship with harmonic eigenvalues, with a roughly exponential function with some deviations. Across all four bands, it is evident that the participation energy curves obtained from SGM-SBI model-predicted FC closely approximate those derived from empirical FCs—a clear validation of the graph harmonic model’s ability to accurately approximate empirical FCs. Interestingly, the model FC’s participation energy curve suggests that it requires a few more harmonics, up to four or five, to capture overall FC energy. Nonetheless, a small fraction of harmonics are sufficient to capture most of the participation energy of model-predicted FC. Both empirical and model FC appear to be dominated by the first three graph harmonics. It is remarkable that a combination of these three canonical structural harmonics are capable of capturing most of the participation energy of both empirical MEG and model-predicted FC.

### 4.5 Empirical and fitted model’s FC matrices

The mean FC matrices across all subjects, obtained via MNE-Connectivity toolbox from MEG time series using the absolute coherence method (seeSection 3.2) for all four bands, are illustrated inFigure 3(middle column) as matrix heatmaps. Each matrix has rows and columns corresponding to brain regions, and elements of the matrix represent the functional connectivity between the two corresponding regions. Empirical FC in all bands appears largely consistent, each displaying strong intrahemispheric but commensurately weaker interhemispheric coherence. There is evidence of modular structure within each hemisphere as well. Note that the diagonal was removed for visualization, since self-connectivity is trivially and always equal to 1 in FC by definition. The frequency bands are defined asdelta, [2, 3.5] Hz,theta, [4, 7] Hz,alpha, [8, 12] Hz, andbeta, [13, 20] Hz.

**Fig. 3.**
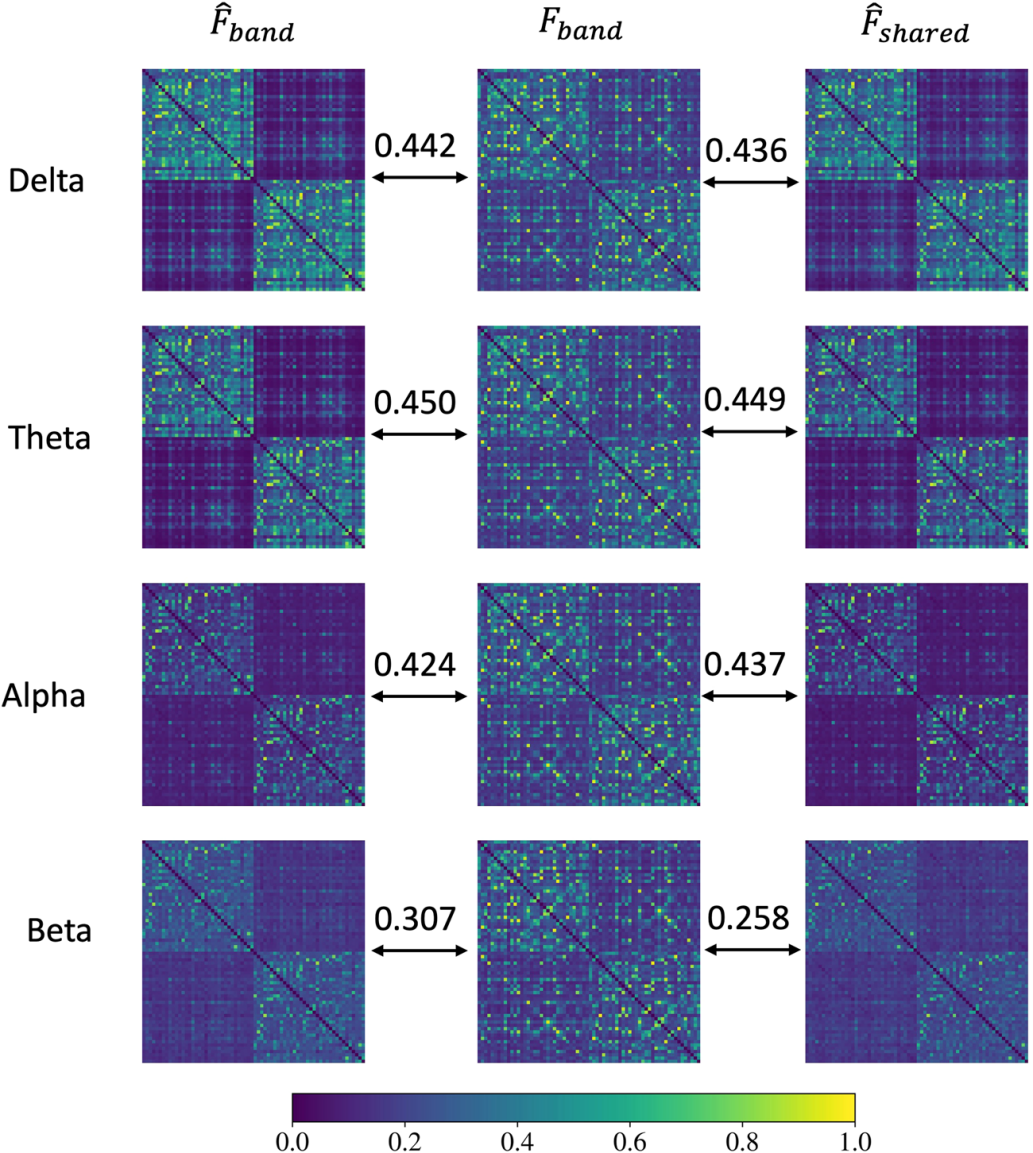
Left column: Mean estimated FCs fordelta,theta,alpha, andbetabands derived from band-specific SGM parmaeters (SGM-SBI); middle column: Mean empirical FCs fordelta,theta,alpha, andbetabands across all subjects; right column: Mean estimated FC fordelta,theta,alpha, andbetabands using band-shared SGM parameters (SGM-SBI-shared). The numbers above the arrows represent the average Pearson’s correlation between estimated and empirical FCs.

For each band and each subject, we fitted the theoretical SGM to the empirical model to obtain the optimal band-specific SGM parameters$\left\{\tau_{\text{g}},v,\alpha \right\}$. These parameters were then used to calculate the estimated FCs viaEquation 3. The left column shows the mean estimated FC obtained from all subjects. Similar to the empirical FCs results, the estimated FCs with band-specific parameters exhibit a consistent pattern across different bands, that is, higher connectivity within hemispheres and lower connectivity between hemispheres. However, when compared with empirical FCs, the estimated FCs have stronger connectivity within hemispheres while weaker connectivity between hemispheres. Moreover, as the frequency of the band increases, there is a significant decrease in the overall density of the estimated FC. This could be partially attributed to the SGM’s design, wherein no attempt was made to capture higher frequencies in the highbetaandgammabands, which are thought to arise from local oscillatory processes that are not directly modulated by the whole brain connectome (Jin et al., 2023;Raj et al., 2020;Verma et al., 2022).

We also explored a variant of SGM-SBI, where we fitted the theoretical FCs from all four bands with a single set of band-shared SGM parameters for each subject. The resulting FCs, referred to as SGM-SBI-shared, are depicted in the rightmost column. The estimated FCs from band-shared parameters closely resemble those from band-specific ones across all four bands. This implies that a single set of SGM parameters can successfully duplicate the MEG FCs across all four frequency bands, suggesting, uniquely in the literature on model-based fitting of MEG, that FC in all bands may be governed by the same biophysical process with the same characteristic parameters. The average Pearson’s correlation between estimated and empirical FCs is displayed inFigure 3. FCs from band-specific and band-shared parameters have comparable Pearson’s correlation with the empirical ones indelta,theta, andalphabands. However, inbetaband, SGM-SBI performs notably better than SGM-SBI-shared.

### 4.6 Posterior distributions of fitted parameters

Now we showcase a key feature of the proposed SGM-SBI framework: its ability to produce the posterior distributions of SGM parameters. After the SBI-based fitting procedure on individual subjects, we draw 1000 samples for each parameter and frequency band from every subject, pooling together the samples from all 36 subjects. These posterior distributions are illustrated inFigure 4, where each subplot shows the univariate densities for each SGM parameter and all MEG bands. For enhanced visualization, each density curve is normalized to have the same peak value. Both versions of the model, SGM-SBI and SGM-SBI-shared, were fitted separately and are shown in the figure.

**Fig. 4.**
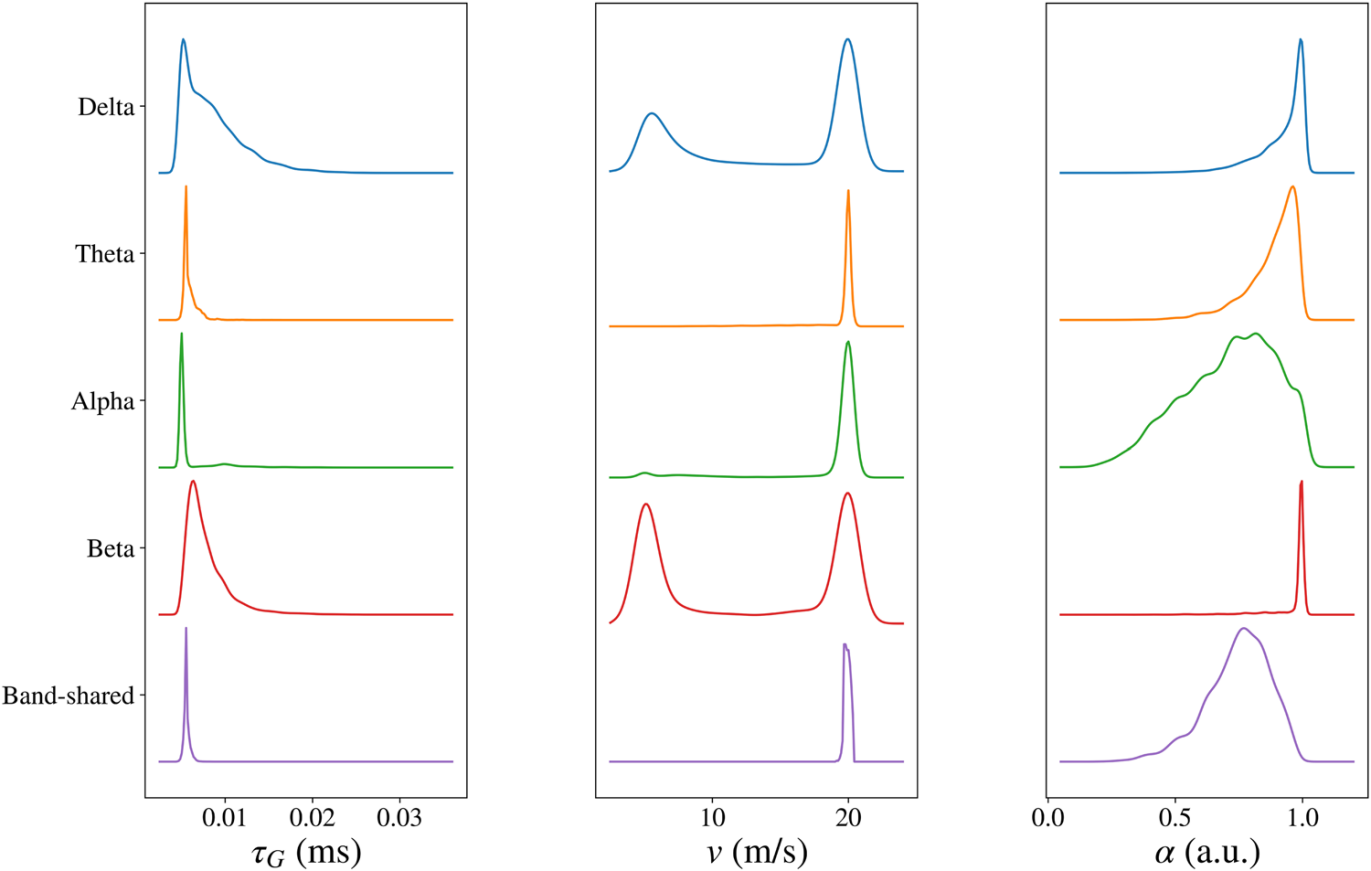
The posterior density plots of the SGM parameters estimated by fitting empirical FCs with SGM-SBI fordelta,theta,alpha, andbetabands and with SGM-SBI-shared method via stacking FCs from four bands together. The SGM parameters include graph time constant$\tau_{\text{g}}$(left), transmission speedv(middle), and coupling constantα(right). Note that these density plots are based on data pooled from all 36 individuals. For better visualization, all the density curves are normalized such that they have the same peak value.

In the left column ofFigure 4, the density plots of four band-specific and one band-shared results for the graph time constant$\tau_{\text{g}}$are displayed. Across all cases, the posterior distributions of$\tau_{\text{g}}$exhibit a similar pattern with a single peak around0.005ms. However, it is important to note that densities of$\tau_{\text{g}}$from bothdeltaandbetabands show greater dispersion compared with others. The results of axonal conductance speedvare shown in the middle column ofFigure 4. The densities fromthetaandalphabands and the band-shared results are all single peaked and concentrated around20m/s. In contrast, for thedeltaandbetabands, the distributions display two distinct peaks situated around 5 and 20 m/s. The results for the global coupling constantαare presented in the right column ofFigure 4. When compared with densities for the time constant$\tau_{\text{g}}$, those forαexhibit a single peak situated approximately at 0.9 a.u., albeit with greater dispersion overall. The densities derived fromdeltaandbetabands demonstrate higher concentration than others. The band-shared results appear to represent a consensus of the parameter posteriors across all bands—while this is to be expected, it points to a possible disambiguating effect of the shared model compared with the fitting on individual bands. We observe that the densities derived from band-shared results are closer to those from thealphaband across all three SGM parameters, which indicates the importance ofalphaband in the study of FC for MEG data.

InFigure 4, some of the density plots show double peaks. It is worth noting that the density plots are based on data pooled from all 36 individuals. The double peaks in the density plots may be due to heterogeneity among the subjects. To verify this, we plot the individual density plots for each subject and do not find any double peaks in the density plots. We select two representative subjects and show the individual density plots inFigures S.1 and S.2. Another issue is that some of the density plots peak at the bounds of the parameters. It raises the question, whether the bounds of the parameters inTable 1are too tight. To investigate this, we computed the average MSE across all subjects between the empirical FC and model-predicted FC using the SGM-SBI method for different values of the parameters, well outside their prescribed bounds; these are shown in Figure S.4. We vary the one parameter and fix the other two parameters at their fitted values. In the plot, the red dashed lines indicate the prescribed upper and lower bounds used during SBI inference. The MSE plots show that the optimal values for each parameter are roughly within the prescribed lower and upper bounds. Although the bounds are governed by biological constraints, the fact that the model fits are generally contained within these bounds is reassuring.

### 4.7 Performance of SGM graph harmonic model fitting

We evaluate the performance of SGM-SBI model fitting in comparison with other methods such as connectome-coupled NMM, eigen-mapping, and direct mapping (i.e., similarity between SC and FC). These alternative models were chosen to represent a broad cross-section of recent efforts spanning a wide range of complexity and computational burden. Coupled NMMs are the most complex and expensive, requiring very large simulations, but are considered the most biologically accurate. A direct correlation between SC and FC is clearly the fastest to evaluate and the least complex—while it does not represent any model, it serves as a good baseline expectation of model-based approaches. In between the two extremes, we also evaluated the eigen-mapping model, which captures the essential elements of diffusive spread of activity on structural graphs (Abdelnour et al., 2014,^2021^;Deslauriers-Gauthier et al., 2020), has a simple closed-form solution via graph harmonics, but does not give wide-band frequency response. There are several versions of the eigen-mapping method, each with varying number of parameters. Here we chose one of the simplest, but the performance of alternative eigen models was quite similar.

Detailed descriptions of these methods are available inSection 3.5and performance metrics are outlined inSection 3.4, which include MSE, Pearson’s correlation, and Lin’s correlation. We derive FC from each model, transform the vectorized FC matrix using the minmax operator, and then compute the metrics. These metrics evaluate in different ways the models’ ability to replicate empirical FCs.

**Fig. 5.**
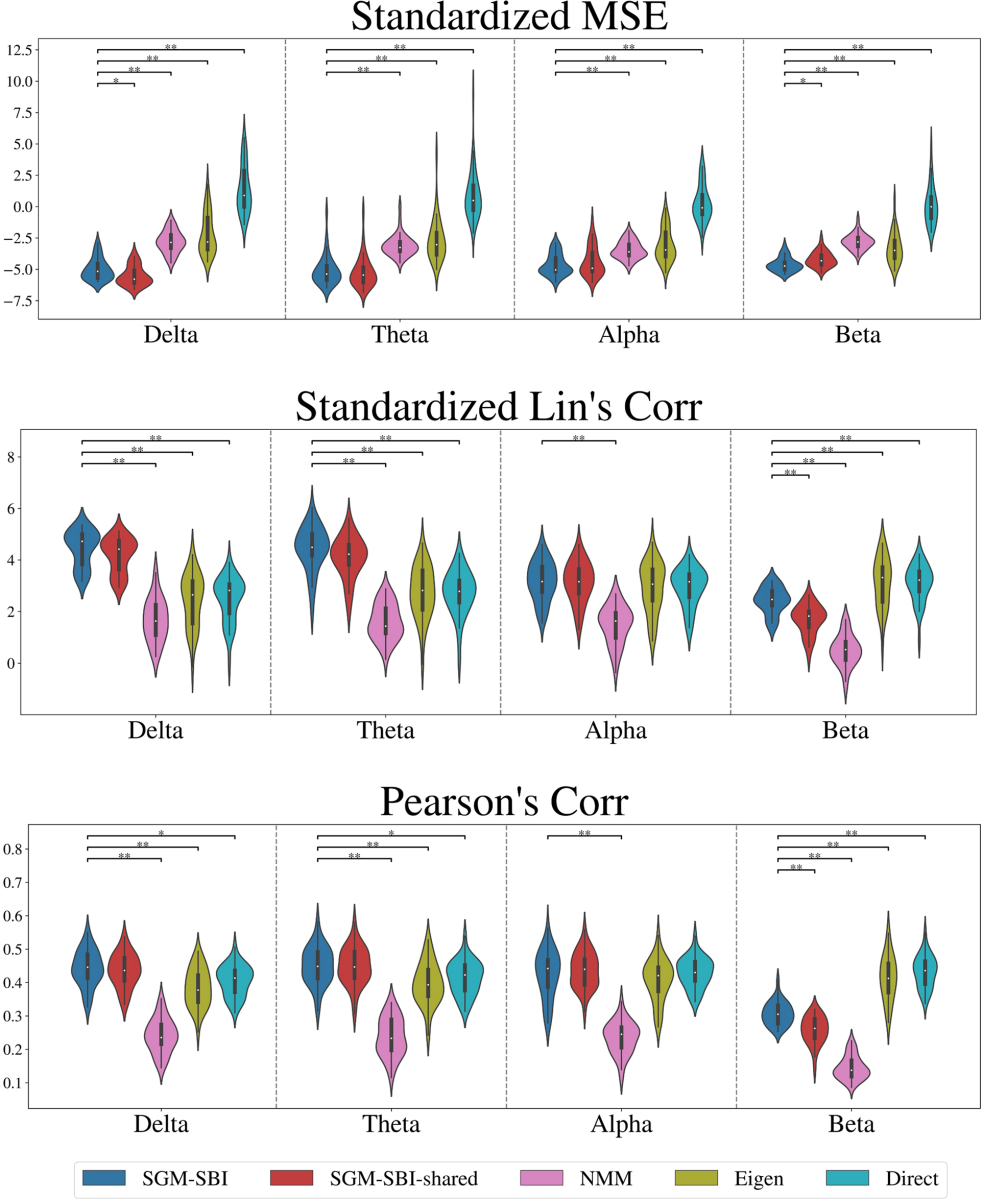
Violin plots of standardized MSE (top), standardized Lin’s correlation (middle), and Pearson’s correlation (bottom) between the empirical FC and model-predicted FC using five competing models: SGM-SBI, SGM-SBI-shared, NMM, eigen-mapping, and direct mapping fordelta,theta,alpha, andbetabands. We aim to assess the performance of different methods on reproducing the empirical MEG FC matrices and use three metrics, MSE (the lower the better), Lin’s correlation (the higher the better), and Pearson’s correlation (the higher the better). Direct mapping refers to the simple correlation or MSE between SC and FC. We show the significant Student’s*t*-test results comparing SGM-SBI with other methods. “*” indicates the test result with a p-value within[0.001,0.05]and “**” indicates the test result with a p-value<0.001.

Figure 5presents the violin plots of MSE (top row), Lin’s correlation (middle row), and Pearson’s correlation (bottom row) under SGM-SBI, SGM-SBI-shared, NMM, eigen-mapping, and direct mapping. For easier interpretation, we standardize the MSE and Lin’s correlation via subtracting the mean and dividing by the standard deviation of the corresponding metrics from spinning the corresponding ROIs of the empirical FCs on the brain surface. By doing so, we create a null model which preserves the spatial structure of the FCs but randomizes the values (Alexander-Bloch et al., 2018). We maintain Pearson’s correlation at the original scale as it has good interpretation. The violin plots of raw MSE, Lin’s correlation, and the standardized Pearson’s correlation are shown in Figure S.3 of theSupplementary Material. We conduct Student’s*t*-test comparing SGM-SBI with other methods on these three metrics and present the significant results inFigure 5, “*” indicates the test result with a p-value within[0.001,0.05]and “**” indicates the test result with a p-value<0.001. For all three metrics, SGM-SBI is generally on par with SGM-SBI-shared indelta,theta, andalphabands which is in agreement with the observation inSection 4.5. In terms of the MSE metric, the SGM-SBI-shared shows some superiority while band-specific fitting performs better in correlation metrics. The distinction between band-specific and band-shared fitting becomes more noticeable in thebetaband where SGM-SBI consistently surpasses SGM-SBI-shared across all metrics. Interestingly, the performance of SGM methods decreases as the band frequency increases. This decline is particularly noticeable in thebetaband, indicating an inability of SGM to handle high-frequency bands. TakingFigures 3,4, and5together, we note again that the band-shared model gives roughly similar FC matrix, performance, and parameter posteriors as the band-specific models.

When compared with other methods, our method outperforms all others based on MSE metric across all four bands. Regarding Lin’s correlation and Pearson’s correlation measures, our methods surpass others in bothdeltaandthetabands. Inalphaband, SGM methods, eigen-mapping, and direct mapping have comparable Lin’s correlation and Pearson’s correlation while the NMM shows worst performance. Inbetaband, two naive methods, that is, eigen-mapping and direct mapping, exhibit highest correlations and SGM methods still outperform NMM. The results show strong correlations but high MSE between empirical FCs and SCs. This implies that while the SC can capture the overall changing trend in FC, it does not provide valuable insights into the magnitude of FC. It is also possible that Pearson correlation may be less informative as a performance metric than the other metrics, likely due to its insensitivity to scale.

#### 4.7.1 Execution time comparison

In addition to visual resemblance and numerical performance of each model, the execution time required to achieve those results is also a matter of high practical impact. We, therefore, compare the computation time of two modeling methods, SGM-SBI and coupled NMM. In a machine with Intel Xeon W-2255 CPU, SGM-SBI takes 119.119 seconds to fit the model to a single subject’s MEG data, and 0.426 second to get 1000 samples on average for thealphaband. Using the same machine, band, and subjects, the NMM model takes approximately an average of 1849.814 seconds to complete fitting using the evolutionary algorithm and produce final results. The SGM-SBI is superior in terms of computational efficiency compared with NMM. While 2 minutes per subject is not quite instantaneous, it is in practice nearly so. The NMM simulations in contrast take around 30 minutes per subject—this is a vast improvement over prior iterations of NMM methods, yet its execution time may not be considered practical in some settings.

### 4.8 Performance of model fitting on regional level

We evaluate the performance of model fitting for SGM-SBI and SGM-SBI-shared at the regional level by calculating the Pearson’s correlation between the corresponding columns of the empirical and estimated FC matrices. In Panel A ofFigure 6, we display the mean Pearson’s correlation between the empirical and estimated FCs for each ROI across all subjects indelta,theta,alpha, andbetabands for both SGM-SBI and SGM-SBI-shared. SGM-SBI and SGM-SBI-shared show similar patterns across all bands. Indelta,theta, andalphabands, the mean Pearson’s correlation is above0.5for most ROIs, while forbetaband, the mean Pearson’s correlation is below0.5for most ROIs. Another observation is that across the brain, the mean Pearson’s correlation is higher in parietal and temporal lobes compared with other regions. We also conduct the one-sample*t*-test versus0for the Pearson’s correlation for each ROI and the negative log p-values are shown in Panel B ofFigure 6. To have a meaningful test, we standardize the Pearson’s correlation via subtracting the mean and dividing by the standard deviation of the Pearson’s correlation from spinning the corresponding ROIs of the empirical FCs on the brain surface as we do inSection 4.5. The cutoff value is set to− log(0.05​/​68)≈7.2after Bonferroni correction and only significant results are shown. Indelta,theta, andalphabands, most of the ROIs have significant Pearson’s correlation, while forbetaband, most of significant ROIs are located in the parietal and temporal lobes.

**Fig. 6.**
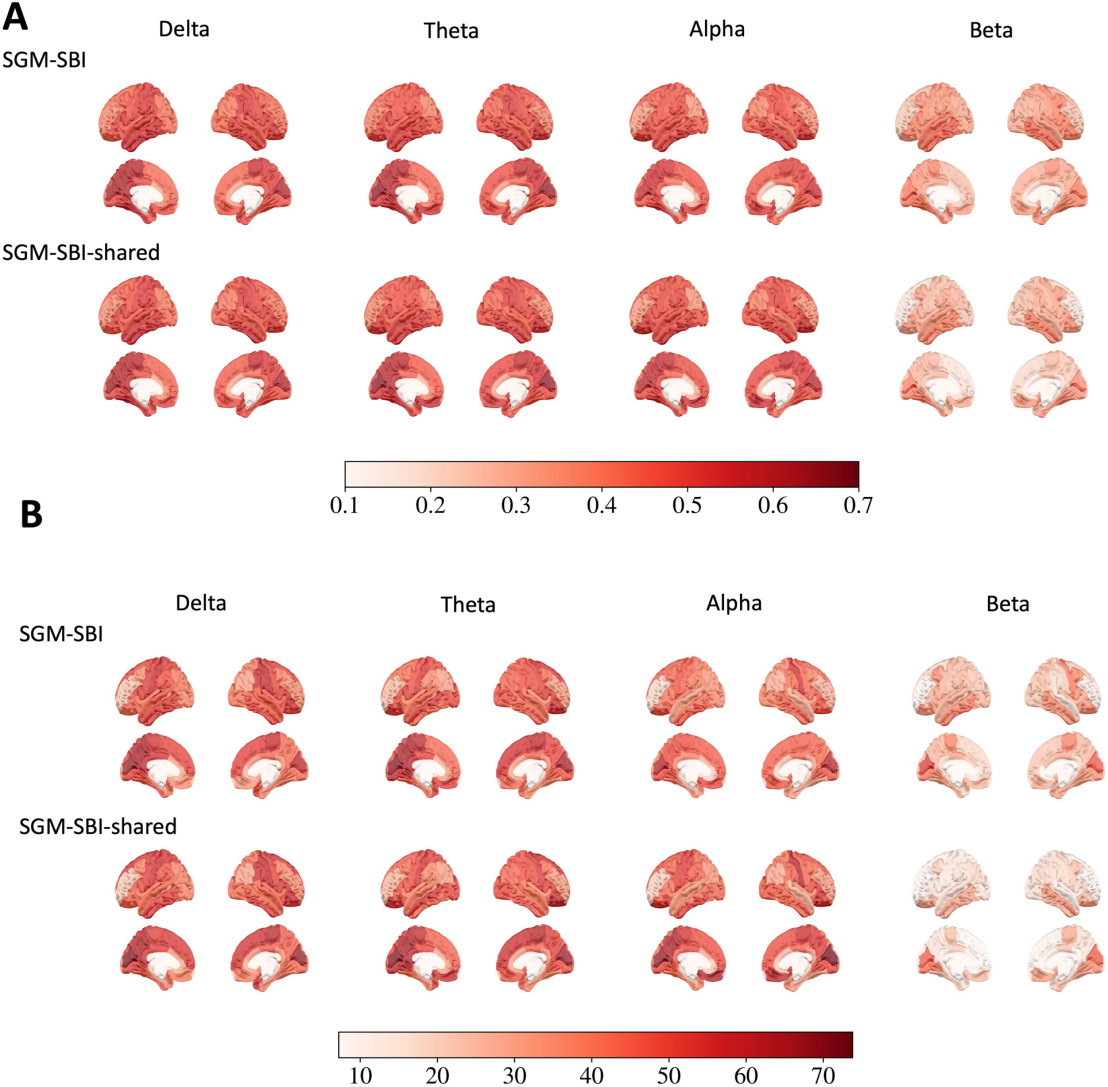
The results of the regional level evaluation for SGM-SBI and SGM-SBI-shared. (A) The mean Pearson’s correlation between the empirical and estimated FCs for each ROI across all subjects indelta,theta,alpha, andbetabands. (B) The negative log p-values of the one-sample*t*-test versus0for the Pearson’s correlation for each ROI across all subjects indelta,theta,alpha, andbetabands. The cutoff value is set to−log(0.05​/​68)≈7.2after Bonferroni correction and only significant results are shown.

### 4.9 Individual-level results

We select two representative subjects to show the individual-level results. Specifically, we choose the subjects with the Pearson’s correlation between the empirical FC and model-predicted (SGM-SBI model) FC closest to the mean of all subjects onalphaband. The results are shown inFigures S.1 and S.2.

For both subjects, the real part of the first three complex Laplacian eigenvectors in thealphaband (Panel A ofFigs. S.1 and S.2) is very similar to the mean eigenvectors across all subjects (Fig. 2A). The estimated FCs from two subjects are also consistent with the mean estimated FCs across all subjects (Fig. 3). In terms of the posterior distributions of the SGM parameters, the group-level results show some bi-modal distributions for transmission speedvindeltaandbetabands (Fig. 4). In the individual-level results, the posterior distributions only show a single peak for all three SGM parameters in all bands (Panel B ofFigs. S.1 and S.2). Though, the peak locations for the transmission speedvindeltaandbetabands are different between the two subjects.

## 5 Discussion

### 5.1 Summary and significance of key findings

In this work, we advance the emerging concept of structural connectome (SC) graph harmonics in the brain by imbuing them with biophysical mechanisms and by demonstrating that a parsimonious combination of harmonics is capable of recapitulating frequency band-specific FC of MEG recordings. The study fills a critical gap in the field, wherein harmonics have been shown to possess predictive power for capturing FC but have not been shown to possess biophysical meaning, while biophysically driven computational models do not have direct involvement of graph harmonics. Our goal was to combine both the elegance of graph harmonics and the biophysical relevance of detailed NMMs. We achieved this goal via a biophysical linearized model of the propagation of brain activity on SC, leveraging and extending advances in modeling wide-band MEG power spectra using graph eigenspectra (Raj et al., 2020;Verma et al., 2022). We provided for the first time a fully frequency-resolved description of FC, at any arbitrary frequency. This description is direct and does not require either simulations in time or indirect calculation of FC via the narrow-band Hilbert envelope (Cabral et al., 2014;Tewarie et al., 2022).

The resulting graph model has only three global and invariant parameters, each with a well-understood biological meaning: characteristic time scale of long-range projection neurons$\tau_{G}$(unit: ms), axonal conductance speedv(m/s), and the connectome coupling constantα(unitless). It is, therefore, noteworthy that empirical FC, which has a prominent spatial organization, can be successfully predicted by a model consisting of only three spatially invariant parameters. The implication is that spatial variance in FC is directly a consequence of the spatial distribution of the low graph harmonics of SC. Note that local model parameters, which were critical in prior fitting of SGM to wide-band spectra, are effectively removed in the current context following normalization of the SGM covariance matrix by its diagonal. This makes intuitive sense, since local processes may affect local spectral power but not long range synchrony between distant regions.

In order to convert these theoretical advances into a practical tool, we designed a speedy and flexible deep learning network for inferring the biophysical parameters. After the network had been trained on sufficient simulation-based training samples, it could achieve model parameter inference almost instantaneously for a given subject. Parameter inference has historically been an intractable challenge in neural system modeling, and prior coupled NMMs require a combination of hand tuning and grid search (Glomb et al., 2022;Xie et al., 2019). In contrast, we were not only able to obtain best-fit biophysical parameters very quickly (within seconds) but also to give their full posterior distributions and accompanying confidence bounds. We demonstrated this inference ability, critical for potential practical applications of model-based SC-FC inference, on a study of 36 healthy individual subjects’ MEG recordings. With the analytical frequency-resolved description of FC, parameter inference is substantially faster and requires limited memory for model computations. By incorporating the SBI framework for inferring parameters, obtaining the posterior densities of parameters is tractable. This resolves a key intractable challenge inherent in the inference of current coupled NMMs, which require lengthy and massive numerical simulations, and opens the door to future practical applications of model fitting to MEG data. Remarkably, using only the three biophysical parameters and only a few (3–5) graph harmonics, the model is able to reproduce empirical MEG FC in all frequency bands of interest, quantifiably better than all competing benchmark methods we tested. The trained DL-based SBI network gives full posterior distributions and confidence bounds, which are necessary for applications where a measure of uncertainty is helpful.

Finally, we explored an important issue in prior modeling studies on MEG: would the model parameters be frequency band specific, or global for a given subject? Most prior successes in theoretical fitting of MEG data appear to be band specific, implying very different biophysical properties in different bands. This is long understood to be biologically implausible, since the parameters pertain to biological systems and should not vary across frequencies. The emergence of the correct frequency dependency should be a key test of a successful computational neural model, a test that is rarely passed in existing literature. Our demonstration that a single parametrization of the harmonic model, that is, a unique combination of parameters$\tau_{G},v,\alpha$, is capable of reproducing MEG FC in all frequency bands of interest, gives renewed assurance that our computational model passes this test of plausibility. Indeed, we were able to show that the band-shared model achieved similar FC matrix visualization (Fig. 3), parameter posteriors (Fig. 4), and numerical performance metrics (Fig. 5) when compared with SGMs fitted to each frequency band separately. If anything, the shared model showed evidence of superior disambiguation and consensus among bands.

### 5.2 Proposed model is tractable compared with coupled neural mass simulations

We numerically compared (Fig. 5) the proposed SGM-SBI model with a current and open-source implementation of NMM simulations. Across all results we evaluated, the connectome-coupled NMM was consistently one of the least accurate predictors of FC in all bands. This might appear surprising at first, given the deep and wide popularity of NMMs in the field, their long history, and their ability to accommodate biological processes of high complexity. It is, therefore, useful to understand the issues that might impact the accuracy and inference performance of these methods.

While coupled NMMs have been widely used to capture MEG FC, they suffer from some challenges that can make robust inference of model parameters intractable, discussed in detail previously (Raj et al., 2022;Xie et al., 2019). First, NMMs simulations take a long time and compute power since they require numerical integration of coupled differential equations. Second, since these nonlinear model solutions can be vastly different depending on the parameter regime, certain model parameters are hand tuned to be near a bifurcation point and only the remaining parameters are optimized for by fitting to the empirical data, typically using a grid search optimization (Breakspear, 2017;Sanz-Leon et al., 2015). Lastly, most prior NMMs studies have focused on fitting to group-level FC (Cabral et al., 2014;Deco et al., 2017;Nakagawa et al., 2014;Tewarie, Abeysuriya, et al., 2019) with fewer studies focused on fitting to individual-level FC (Abeysuriya et al., 2018;Kulik et al., 2023;Tewarie, Hunt, et al., 2019). SGM is able to address all of these challenges given its analytic closed-form solution consisting of only a few global parameters, and its ability to fit to individual-level FC fast with the SBI framework, taking 2 minutes compared with 30 minutes by NMM.

A key point to note is that most neural mass models are nonlinear and can, therefore, exhibit a rich dynamical repertoire in their oscillatory behavior (Cabral et al., 2014,^2017^;Siettos & Starke, 2016). Such behaviors are quantified in terms of bifurcations defining solution regimes that are quantified by fixed points, limit cycles, and chaotic behavior. Since the current graph model is linear, it can only exhibit stable or oscillatory, but no chaotic behavior (Verma et al., 2023). It is still an open question whether such nonlinearities are required to capture macroscopic structure–function relationships. Indeed, macroscopic spatial and frequency patterns are largely identical across individuals (Freeman & Zhai, 2009;He et al., 2010;Robinson et al., 2005). It has been suggested that emergent long-range activity can be independent of microscopic local activity of individual neurons (Abdelnour et al., 2014;Destexhe & Sejnowski, 2009;Mišić et al., 2014,^2015^;Robinson et al., 2005;Shimizu & Haken, 1983), and that these long-range activities may be regulated by the long-range connectivity (Abdelnour et al., 2015;Deco et al., 2012;Jirsa et al., 2002;Nakagawa et al., 2014). In addition, linear models outperformed nonlinear models in predicting resting-state fMRI time series (Nozari et al., 2020). Therefore, to capture macroscopic phenomena, the present deterministic graph model may be sufficient, with the additional advantage of tractable model inference.

### 5.3 Alternative inference methods

In prior studies that use a similar spectral graph model (SGM) (Raj et al., 2020,^2022^;Verma et al., 2022,^2023^), annealing-based optimization was used for inference, which suffers from two limitations. First, it can only provide a single point estimate of the parameters. In a neural model like SGM, it is desirable to find out not only the best, but also all parameter settings, compatible with the observed data. The variability of the parameters under the observation can provide more insights about the neural models and processes (Alonso & Marder, 2019;Gonçalves et al., 2020). Annealing optimization fails to meet this requirement. Moreover, it is difficult to incorporate prior knowledge about neural processes with annealing, which further limits its application. The free parameters in neural models typically relate to biological processes, hence their inference must utilize constraints that avoid unreasonable solutions. Using prior knowledge of these biological quantities can not only increase optimization efficiency but also make them more robust.

It is also possible to use Markov chain Monte Carlo (MCMC) methods (Raftery & Lewis, 1996) to conduct Bayesian inference, as our model has a closed-form solution in the Fourier frequency domain. However, the posterior densities are challenging to analyze, a long burn-in step is required for MCMC methods to reach the equilibrium distribution, and samples from the equilibrium distribution are correlated. Thus sampling from MCMC can be time consuming for SGM. Previously, an MCMC-based inference was unable to capture the spectral features using a nonlinear neural mass model (Xie et al., 2019). In comparison, SBI is more flexible and can handle complicated likelihood functions. More importantly, SBI is trained upfront with simulation samples, which helps to reduce the requirements of real data and greatly improves inference speed.

Dynamic causal modeling (DCM) (Kiebel et al., 2008;Pinotsis et al., 2012) is a Bayesian approach for estimating and comparing models of functional signals (e.g., fMRI, MEG, or EEG time series). The activities of neuronal populations are modeled by systems of differential equations representing synaptic coupling and its plasticity. The “hidden” neuronal dynamics are linked to measured time series data through a biologically grounded forward model. Recent work on regression DCM (Frässle et al., 2017,^2018^) proposes local neural mass models formulated in the steady state frequency domain, with the effective connectivity evaluated from the cross spectra. Such models assume numerous degrees of freedom, while the present SGM requires only three global parameters. Hence there is a key difference in the goals and outcomes of DCM and the present study: the former seeks to estimate the entire matrix of pairwise associations from the time series data, whereas the latter seeks to enforce SC as the basis for predicting FC. Another important difference between DCM and SGM is that DCM requires variational Bayesian inference, whereas here we leveraged the SBI tool for posterior inference of the global SGM parameters with ranges based on underlying biophysics.

### 5.4 Limitations

In this work, FC was constructed using absolute coherence, while other groups have favored partial coherence (Wodeyar & Srinivasan, 2022). While we enforced spatial uniformity in model parameters, it is possible that some spatial heterogeneity may help improve model fits—an aspect we will explore in the future. Specifically, it might be necessary to accommodate the variation of the speed parameter as a function of the amount of myelination and synaptic strength in different regions. Various methodological limitations are relevant here. Our structural connectivity network was obtained based on diffusion-weighted images which are only an approximation of white-matter axonal connections. The MEG functional network was source reconstructed using a minimum-variance adaptive beamformer, a process which may be ill-posed and one that has difficulty in estimating deep brain sources (Hillebrand & Barnes, 2005;Henson et al., 2009). Despite these approximations, macroscopic spatial and frequency patterns are largely robust across individuals (Freeman & Zhai, 2009;He et al., 2010;Robinson et al., 2005). We have discussed these in detail in prior works (Raj et al., 2022;Xie et al., 2021). Our model’s FC output is directly comparable with coherence-based FC, due to the connection with cross-spectral density. The relevance of our model in producing other types of FC, for example, amplitude envelope correlation, is unclear at this time, and will be explored in future work.

Another limitation is the assumption, noted inSection 2.2, that*the driving input signal is uncorrelated across regions and across time*. We assume for simplicity that$\varepsilon \left(\text{P}(\omega )\;\text{P}{\left(\omega \right)}^{H}\right)$has independent and identically distributed white spatial and temporal covariance structure. This assumption greatly simplifies the mathematics and the final expression for the model-predicted spatial covariance. However, in some instances, even at rest, the driving function may have a specific spatial covariance structure, such as those related to thalamocortical drive or correlated sensory or motor signals. We plan to explore the influence of such spatial covariance structures in future work. In contrast, temporal covariance in the driving function will only result in frequency-specific scaling of the model covariance, altering predictions but not the overall model architecture.

Furthermore, our model and empirical FC calculations do not consider the role of spatial leakage which is considered a major confound in MEG FC measures, especially at voxel level imaging. Incorporating spatial leakage in model FC calculations to match with empirical FC that account for spatial leakage is something we will pursue in the future.

### 5.5 Potential applications

Given the ease of parameter inference, this work can be readily extended to investigate biophysical alterations resulting in abnormal FC in diseases, such as in Alzheimer’s disease (Alonso et al., 2011;Escudero et al., 2011;Franciotti et al., 2006;Ranasinghe et al., 2014), epilepsy (Li et al., 2022), addiction (Racine et al., 2017), and multiple sclerosis (Lassmann & Bradl, 2017), to name a few. It can also be used to examine underlying biophysical mechanisms that shape FC in processes such as cognition (Beppi et al., 2021). Since the proposed graph model parameters have biophysical interpretability, inferring them can suggest meaningful biophysical alterations in the excitatory and inhibitory neuronal populations that can result in changes in FC. Previously, we have shown such alterations at a local level in Alzheimer’s disease by investigating the empirical MEG spectra (Ranasinghe et al., 2022). Inferring such biophysical mechanisms is unfeasible with examining raw neuroimaging data alone, or with models where parameter inference is intractable. Additionally, the posterior densities of model parameters provide us with error bounds that can be useful for clinical applications, specifically for identifying biophysical markers of disease. In the future, we will also aim at simultaneously capturing both FC and power spectra using graph harmonics, for example,Raj et al. (2020). This will aid in identifying common underlying biophysical mechanisms that shape both spectra and FC.

## Supplementary Material

Supplementary Material

## Data Availability

The code and processed datasets for this work can be found in this github repository:https://github.com/JINhuaqing/SBI-SGM-FC_paper. OtherSupplementary Materialsare available online.
