# Supplementary material for: Bayesian inference of frequency-specific functional connectivity in MEG imaging using a spectral graph model
Source: Imaging Neurosci (Camb). 2024 Oct 10;2:imag-2-00307. doi: 10.1162/imag_a_00307 (PMC12290874; doi:10.1162/imag_a_00307)
Supplement: Supplementary Material [file imag_a_00307-supp.pdf]

Supplementary Materials for "Bayesian Inference of Frequency Specific Functional Connectivity in MEG Imaging Using a Spectral Graph Model"

S.1 Details of Data Processing

MRI A 3 Tesla TIM Trio MR scanner (Siemens, Erlangen, Germany) was used to perform MRI using a 32-channel phased-array radiofrequency head coil. High-resolution MRI of each subject's brain was collected using an axial 3D magnetization prepared rapid-acquisition gradient-echo T1-weighted sequence (echo time [TE] = 1.64 ms, repetition time [TR] = 2,530 ms, TI = 1,200 ms, flip angle of 7°) with a 256-mm field of view, and 160 1.0-mm contiguous partitions at a 256 $\times$ 256 matrix. Whole-brain diffusion-weighted images were collected at b = 1,000s/mm^2 with 30 directions using 2-mm voxel resolution in-plane and through-plane.

Region parcellations The T1-weighted images were parcellated into 68 cortical regions and 18 subcortical regions using the Desikan–Killiany atlas available in the FreeSurfer software (Fischl et al., 2002). To do this, the subject-specific T1-weighted images were back-projected to the atlas using affine registration, as described in the previous studies (Abdelnour et al., 2014; Owen et al., 2013).

Structural connectivity networks Different structural connectivity networks were reconstructed with the same Desikan–Killiany parcellations. Firstly, openly available diffusion MRI data were obtained from the MGH-USC Human Connectome Project to create an average template connectome. As in previous studies (Abdelnour et al., 2014; Owen et al., 2013), subject-specific structural connectivity was computed on diffusion MRI data: *Bedpostx* was used to determine the orientation of brain fibers in conjunction with *FLIRT*, as implemented in the *FSL software* (Jenkinson et al., 2012). In order to determine the elements of the adjacency matrix, tractography was performed using *probtrackx2*. 4,000 streamlines were initiated from each seed voxel corresponding to a cortical or subcortical gray matter structure and how many of these streamlines reached a target gray matter structure was tracked. The weighted connection between the two structures $c_{i,j}$, was defined as the number of streamlines initiated by voxels in region i that reach any voxel within region j, normalized by the sum of the source and target region volumes ($c_{i,j} = \frac{streamlines}{v_i+v_j}$). Afterward, connection strengths were averaged between both directions ($c_{i,j}$ and $c_{j,i}$) to form undirected edges. To determine the geographic location of an edge, the top 95% of nonzero voxels by streamline count were computed for both edge directions. The consensus edge was defined as the union between both post-threshold sets.

MEG data MEG recordings were acquired at UCSF using a 275-channel CTF Omega 2,000 whole-head MEG system from VSM MedTech (Coquitlam, BC, Canada). All subjects were instructed to keep their eyes closed for 5 min while their MEGs were recorded at a sampling frequency of 1,200 Hz.

MEG processing and source reconstruction MEG recordings were downsampled from 1,200 Hz to 600 Hz, then digitally filtered to remove DC offset and any other noisy artifact outside of the 1 to 160 Hz bandpass range. Since MEG data are in sensor space, meaning they represent the signal observable from sensors placed outside the head, this data needed to be "inverted" in order to infer the neuronal activity that had generated the observed signal by solving the so-called inverse problem. Adaptive spatial filtering algorithms were used from the NUTMEG software tool written in house (Dalal et al., 2004). To prepare for source localization, all MEG sensor locations were co-registered to each subject's anatomical MRI scans. The lead field (forward model) for each subject was calculated in NUTMEG using a multiple local-spheres head model (three-orientation lead field) and an 8 mm voxel grid which generated more than 5,000 dipole sources, all sources were normalized to have a norm of 1. Finally, the MEG recordings were projected into the source space using a beamformer spatial filter. Only the sources belonging to the 68 cortical regions were selected to be averaged around the centroid. All dipole sources were labeled based on the Desikan–Killiany parcellations, then sources within a 20 mm radial distance to the centroid of each brain region were extracted, and the average time course of each region's extracted sources served as empirical resting-state data for our proposed model. MEG recordings were bandpass filtered between 2 to 45 Hz using `firls` in MATLAB (MAT, 2020) and the static frequency PSD was generated for every region of interest using the `pmtm` algorithm in MATLAB (MAT, 2020).

S.2 Spectral Graph Model

Notation All the vectors and matrices are written in boldface and the scalars are written in normal font. The frequency f of a signal is specified in Hertz (Hz), and the corresponding angular frequency $\omega = 2\pi f$ is used to obtain the Fourier transforms. The connectivity matrix is defined as $\mathbf{C} = c_{jk}$, where c_{jk} is the connectivity strength between regions j and k, normalized by the row degree.

Mesoscopic Model

Given region k out of N regions, we denote the local excitatory signal as $x_{\mathrm{e}}(t)$, local inhibitory signal as $x_{\mathrm{i}}(t)$, and the long-range macroscopic signals as $x_k(t)$. Combining the decay of individual signals, coupling of excitatory and inhibitory signals as well as input white Gaussian noise, the evolution models of $x_{\mathrm{e}}(t)$ and $x_{\mathrm{i}}(t)$ are:

$$\frac{\mathrm{d}x_{\mathrm{e}}(t)}{\mathrm{d}t} = -\frac{f_{\mathrm{e}}(t)}{\tau_{\mathrm{e}}} \star \big(g_{\mathrm{ee}}\, x_{\mathrm{e}}(t) - g_{\mathrm{ei}}\, f_{\mathrm{i}}(t) \star x_{\mathrm{i}}(t)\big) + p(t)\,, \text{ and,} \tag{1}$$

$$\frac{\mathrm{d}x_{\mathrm{i}}(t)}{\mathrm{d}t} = -\frac{f_{\mathrm{i}}(t)}{\tau_{\mathrm{i}}} \star \big(g_{\mathrm{ii}}\, x_{\mathrm{i}}(t) + g_{\mathrm{ei}}\, f_{\mathrm{e}}(t) \star x_{\mathrm{e}}(t)\big) + p(t)\,, \tag{2}$$

where $f_{\mathrm{e}}(t)$ and $f_{\mathrm{i}}(t)$ are the ensemble average neural impulse response function, $\star$ stands for convolution, $p(t)$ is input noise, parameters g_{ee}, g_{ii}, g_{ei} are neural gain terms, and parameters τ_{e}, τ_i are characteristic time constants, which are shared for every region k. We assume Gamma-shaped $f_{\mathrm{e}}(t)$ and $f_{\mathrm{i}}(t)$ as

$$f_{\mathrm{e}}(t) = \frac{1}{\tau_{\mathrm{e}}^2} \exp\left(\frac{-t}{\tau_{\mathrm{e}}}\right) \text{ and } f_{\mathrm{i}}(t) = \frac{1}{\tau_{\mathrm{i}}^2} \exp\left(\frac{-t}{\tau_{\mathrm{i}}}\right).$$

Macroscopic Model

Accounting for long-range connections between brain regions, the macroscopic signal x_k is assumed to conform to the following evolution model:

$$\frac{\mathrm{d}x_k(t)}{\mathrm{d}t} = -\frac{1}{\tau_\mathrm{G}} f_\mathrm{G}(t) \star x_k(t) + \frac{\alpha}{\tau_\mathrm{G}} f_\mathrm{G}(t) \star \sum_{j=1}^{N} c_{jk} x_j(t - \tau^v_{jk}) + \big(x_\mathrm{e}(t) + x_\mathrm{i}(t)\big)\,, \tag{3}$$

where, τ_G is the graph characteristic time constant, α is the global coupling constant, c_{jk} are elements of the connectivity matrix, τ^v_{jk} is the delay in signals reaching from the jth to the kth region, v is the cortico-cortical fiber conduction speed with which the signals are transmitted. The delay τ^v_{jk} is calculated as d_{jk}/v, where d_{jk} is the distance between regions j and k and $x_\mathrm{e}(t) + x_\mathrm{i}(t)$ is the input signal determined from Equations (1) and (2). The Gamma-shaped $f_\mathrm{G}(t)$ is written as

$$f_\mathrm{G}(t) = \frac{1}{\tau_\mathrm{G}^2} \exp\left(\frac{-t}{\tau_\mathrm{G}}\right).$$

The neural gain g_ee is kept as 1 to ensure parameter identifiability, therefore, SGM only includes 7 identifiable parameters as listed in Table 1.

Closed-Form Model Solution in the Fourier Domain

A salient feature of SGM is that it provides a closed-form solution of brain oscillations under the frequency domain. Let $\mathcal{F}$ be the Fourier transform at angular frequency $\omega = 2\pi f$. Note that the mesoscopic models for different regions share the same parameters, therefore, without loss of generality, we can drop the subscript k.

The solutions for $x_\mathrm{e}(t)$ and $x_\mathrm{i}(t)$ under the frequency domain are

$$X_\mathrm{e}(\omega) = \mathcal{F}(x_\mathrm{e}(t)) = \frac{\left\{1 + \frac{g_\mathrm{ei} F_\mathrm{e}(\omega) F_\mathrm{i}(\omega)/\tau_\mathrm{e}}{j\omega + g_\mathrm{ii} F_\mathrm{i}(\omega)/\tau_\mathrm{i}}\right\} P(\omega)}{j\omega + g_\mathrm{ee} F_\mathrm{e}(\omega)/\tau_\mathrm{e} + \frac{(g_\mathrm{ei} F_\mathrm{e}(\omega) F_\mathrm{i}(\omega))^2}{\tau_\mathrm{e}\tau_\mathrm{i}(j\omega + g_\mathrm{ii} F_\mathrm{i}(\omega)/\tau_\mathrm{i})}} = H_\mathrm{e}(\omega) P(\omega),$$

and

$$X_\mathrm{i}(\omega) = \mathcal{F}(x_\mathrm{i}(t)) = \frac{\left\{1 + \frac{g_\mathrm{ei} F_\mathrm{e}(\omega) F_\mathrm{i}(\omega)/\tau_\mathrm{i}}{j\omega + g_\mathrm{ee} F_\mathrm{e}(\omega)/\tau_\mathrm{e}}\right\} P(\omega)}{j\omega + g_\mathrm{ii} F_\mathrm{i}(\omega)/\tau_\mathrm{i} + \frac{(g_\mathrm{ei} F_\mathrm{e}(\omega) F_\mathrm{i}(\omega))^2}{\tau_\mathrm{e}\tau_\mathrm{i}(j\omega + g_\mathrm{ee} F_\mathrm{e}(\omega)/\tau_\mathrm{e})}} = H_\mathrm{i}(\omega) P(\omega),$$

where $P(\omega)$, $F_\mathrm{e}(\omega)$, $F_\mathrm{i}(\omega)$, $F_\mathrm{G}(\omega)$ are the Fourier transform of $p(t)$, $f_\mathrm{e}(t)$, $f_\mathrm{i}(t)$, and $f_\mathrm{G}(t)$ at angular frequency ω.

We define the complex Laplacian matrix $\mathcal{L}(\omega) = \mathrm{i} - \alpha \mathbf{C}^*(\omega)$ where $\mathbf{C}^*(\omega) = [c_{ij} \exp(-j\omega\tau^v_{ij})]_{i,j=1,\dots,N}$. The solution of the macroscopic signals at a angular frequency ω is

$$\mathbf{X}(\omega) = [\,\mathcal{F}(x_1(t)), \cdots, \mathcal{F}(x_N(t))]^\mathrm{T} = \left(j\omega + \frac{1}{\tau_\mathrm{g}} F_\mathrm{g}(\omega)\mathcal{L}(\omega)\right)^{-1} H_\mathrm{local}(\omega)\mathbf{P}(\omega), \tag{4}$$

where $H_\mathrm{local}(\omega) = H_\mathrm{e}(\omega) + H_\mathrm{i}(\omega)$.

From here, we can re-write $\mathbf{X}(\omega)$ by using the eigendecomposition of the complex Laplacian matrix $\mathcal{L}(\omega)$ which is:

$$\mathcal{L}(\omega) = \boldsymbol{U}(\omega)\boldsymbol{\Lambda}(\omega)\boldsymbol{U}(\omega)^H, \tag{5}$$

where, $\boldsymbol{U}(\omega)$ are the eigenvectors and $\boldsymbol{\Lambda}(\omega) = \text{diag}([\lambda_1(\omega), \dots, \lambda_N(\omega)])$ consist of the eigenvalues $\lambda_1(\omega), \dots, \lambda_N(\omega)$, at angular frequency ω.

By using the above eigen-decomposition of the Laplacian matrix, the $\mathbf{X}(\omega)$ can be re-written as:

$$\mathbf{X}(\omega) = \sum_{k=1}^{N} \frac{\boldsymbol{u}_k(\omega)\boldsymbol{u}_k(\omega)^{\mathrm{H}}}{\mathrm{j}\omega + \tau_{\mathrm{G}}^{-1}\lambda_k(\omega)F_{\mathrm{G}}(\omega)} H_{\mathrm{local}}(\omega)\mathbf{P}(\omega), \tag{6}$$

where, $\boldsymbol{u}_k(\omega)$ are the eigenvectors from $\boldsymbol{U}(\omega)$ and $\lambda_k(\omega)$ are the eigenvalues from $\boldsymbol{\Lambda}(\omega)$ obtained by the eigen-decomposition of the Laplacian matrix $\mathcal{L}(\omega)$ obtained in Eq. (5). Equation (6) is the closed-form steady state solution of the macroscopic signals at a specific angular frequency ω. As SGM provides a closed-form solution $\mathbf{X}(\omega)$, we can compare the modeled and empirical power spectra to estimate the global parameters.

S.3 More Plots

- **Individual-Level Results:** While in the main text we focus on group-level results, we also provide individual-level results in Figure S.1 and Figure S.2. We select two representative subjects to show the individual-level results. Specifically, we choose the subjects with the Pearson's correlation between the empirical FC and model-predicted (SGM-SBI model) FC closest to the mean of all subjects on $alpha$ band.

- **Other metrics results:** Figure S.3 shows the original MSE, original Lin's correlation and standardized Pearson's correlation between the empirical FC and model-predicted FC as the supplementary for Figure 5.

- **MSE versus parameters:** Figure S.4 shows the average MSE across 36 subjects between the empirical FC and model-predicted FC using the SGM-SBI model for different values of the SGM parameters. For each parameter, we vary it and fix the others to the optimal values obtained from the SBI algorithm. The red dashed lines represent the boundaries we used as indicated in Table 1.

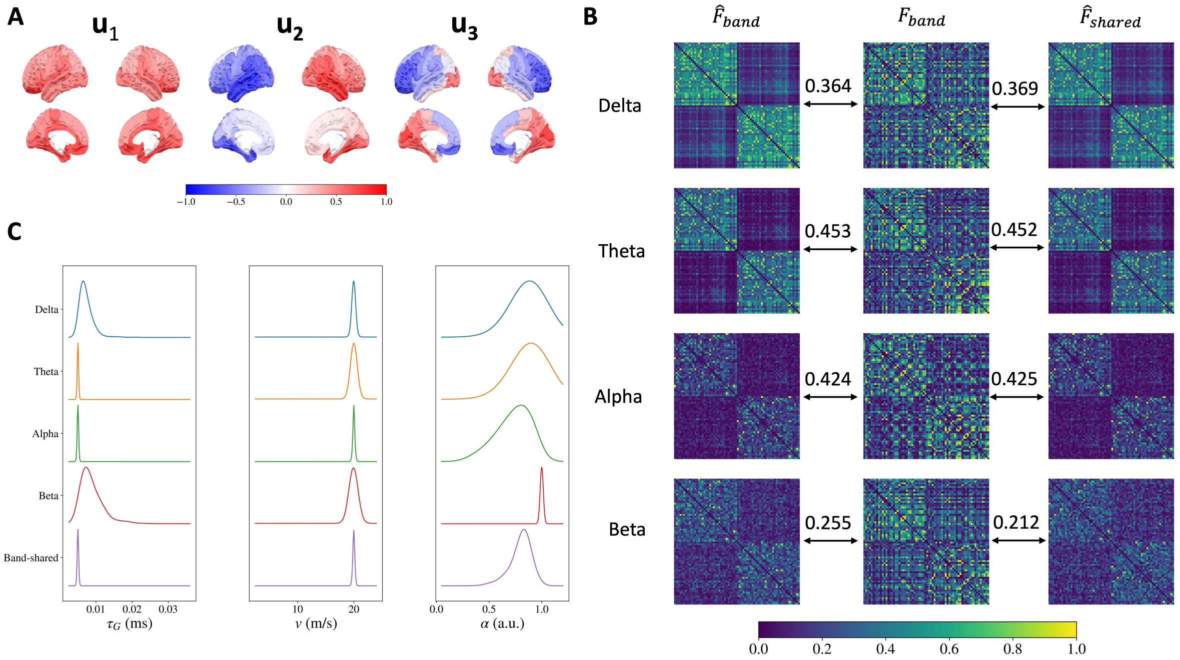

Figure S.1: Results from the first representative subject. **A:** The real part of first three complex Laplacian eigenvectors at the $alpha$ band plotted on the brain. **B:** Mean estimated FCs for $delta$, $theta$, $alpha$ and $beta$ bands from the SGM-SBI model (left), empirical FCs (middle) and SGM-SBI-shared model (right). The numbers above the arrows represent the average Pearson's correlation between estimated and empirical FCs. **C:** The posterior density plots of the SGM parameters estimated by fitting empirical FCs with SGM-SBI for $delta$, $theta$, $alpha$, $beta$ bands and with SGM-SBI-shared method via stacking FCs from 4 bands together. The SGM parameters include graph time constant τ_g (left), transmission speed v (middle) and coupling constant α (right). For better visualization, all the density curves are normalized such that they have the same peak value.

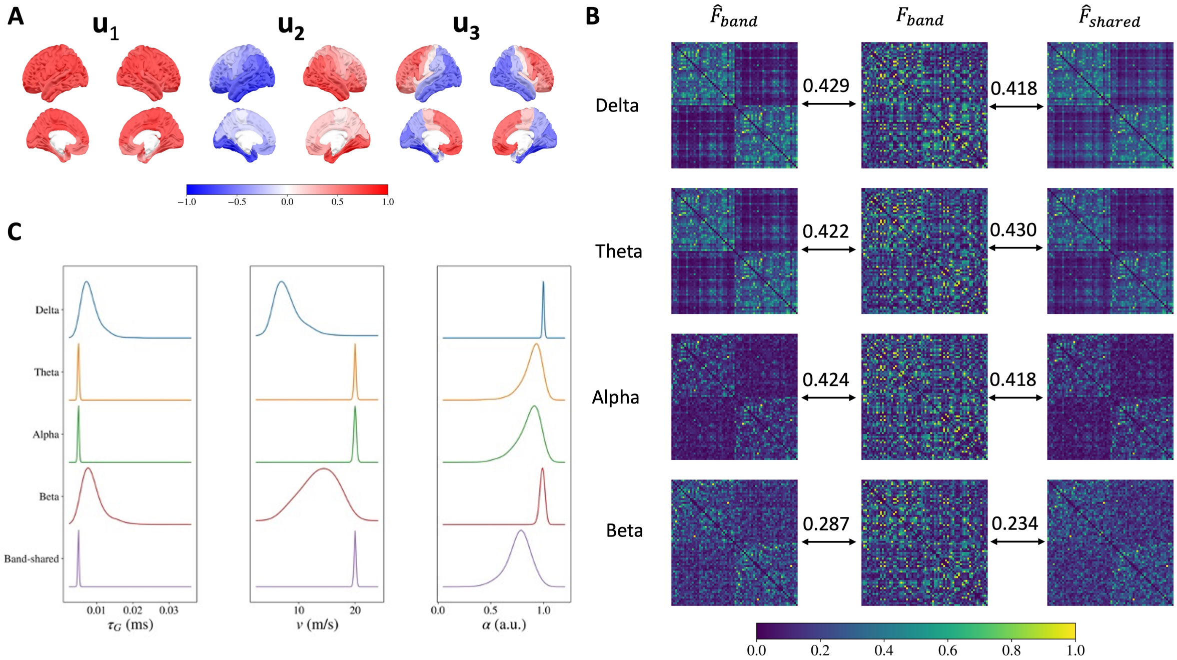

Figure S.2: Results from the second representative subject. **A:** The real part of first three complex Laplacian eigenvectors at the $alpha$ band plotted on the brain. **B:** Mean estimated FCs for $delta$, $theta$, $alpha$ and $beta$ bands from the SGM-SBI model (left), empirical FCs (middle) and SGM-SBI-shared model (right). The numbers above the arrows represent the average Pearson's correlation between estimated and empirical FCs. **C:** The posterior density plots of the SGM parameters estimated by fitting empirical FCs with SGM-SBI for $delta$, $theta$, $alpha$, $beta$ bands and with SGM-SBI-shared method via stacking FCs from 4 bands together. The SGM parameters include graph time constant τ_{g} (left), transmission speed v (middle) and coupling constant α (right). For better visualization, all the density curves are normalized such that they have the same peak value.

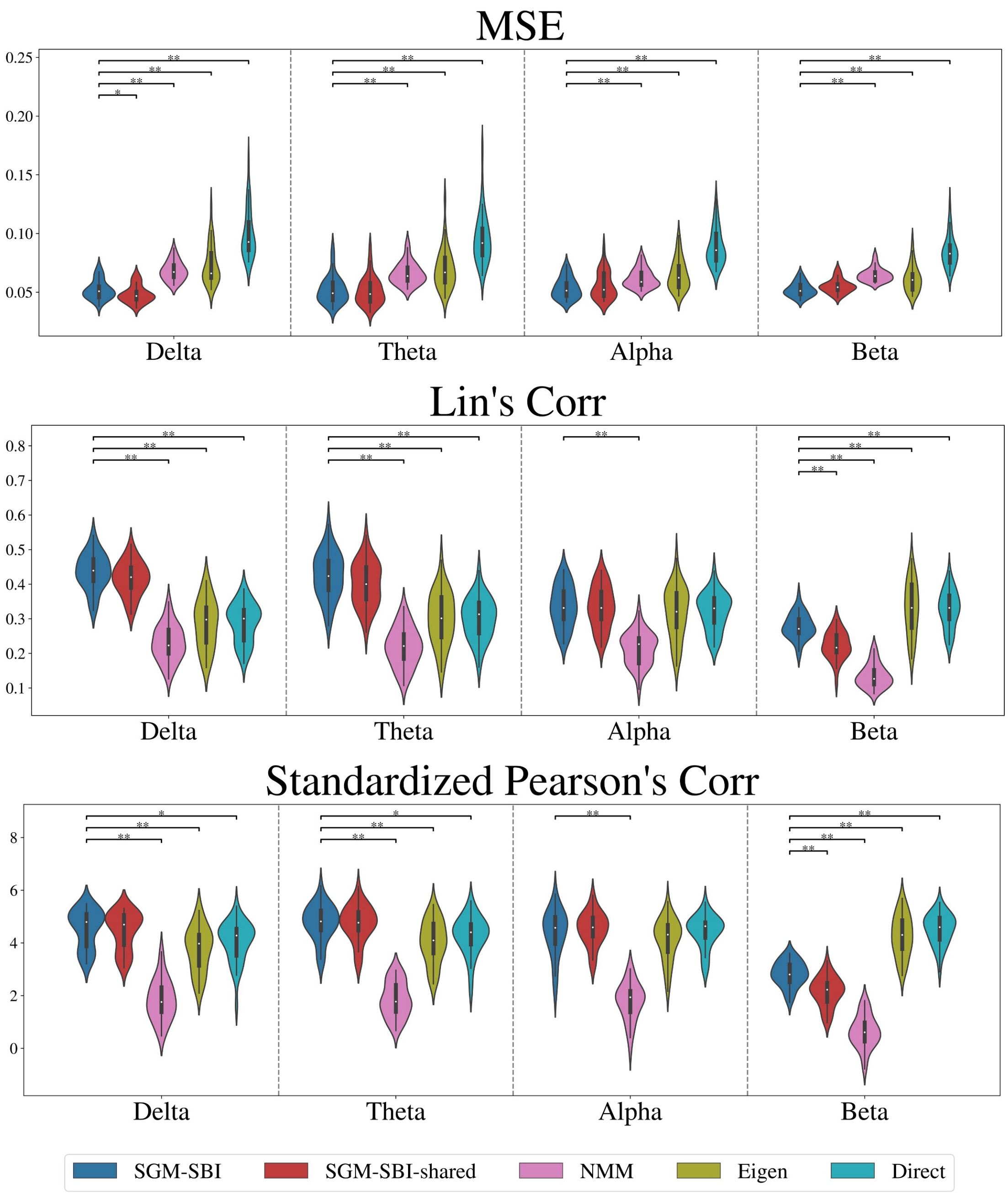

Figure S.3: Violin plots of MSE (top), Lin's correlation (middle) and standardized Pearson's correlation (bottom) between the empirical FC and model-predicted FC using five competing models: SGM-SBI, SGM-SBI-wGeo, SGM-SBI-shared, SGM-SBI-shared-wGeo, NMM, eigen-mapping and direct mapping for $delta$, $theta$, $alpha$ and $beta$ bands. We aim to assess the performance of different methods on reproducing the empirical MEG FC matrices and use three metrics, MSE (the lower the better), Lin's correlation (the higher the better) and Pearson's correlation (the higher the better). Direct mapping refers to the simple correlation or MSE between SC and FC. We show the significant Student's t-test results comparing SGM-SBI with other methods. "*" indicates the test result with a p-value within $[0.001, 0.05]$ and "**" indicates the test result with a p-value < 0.001.

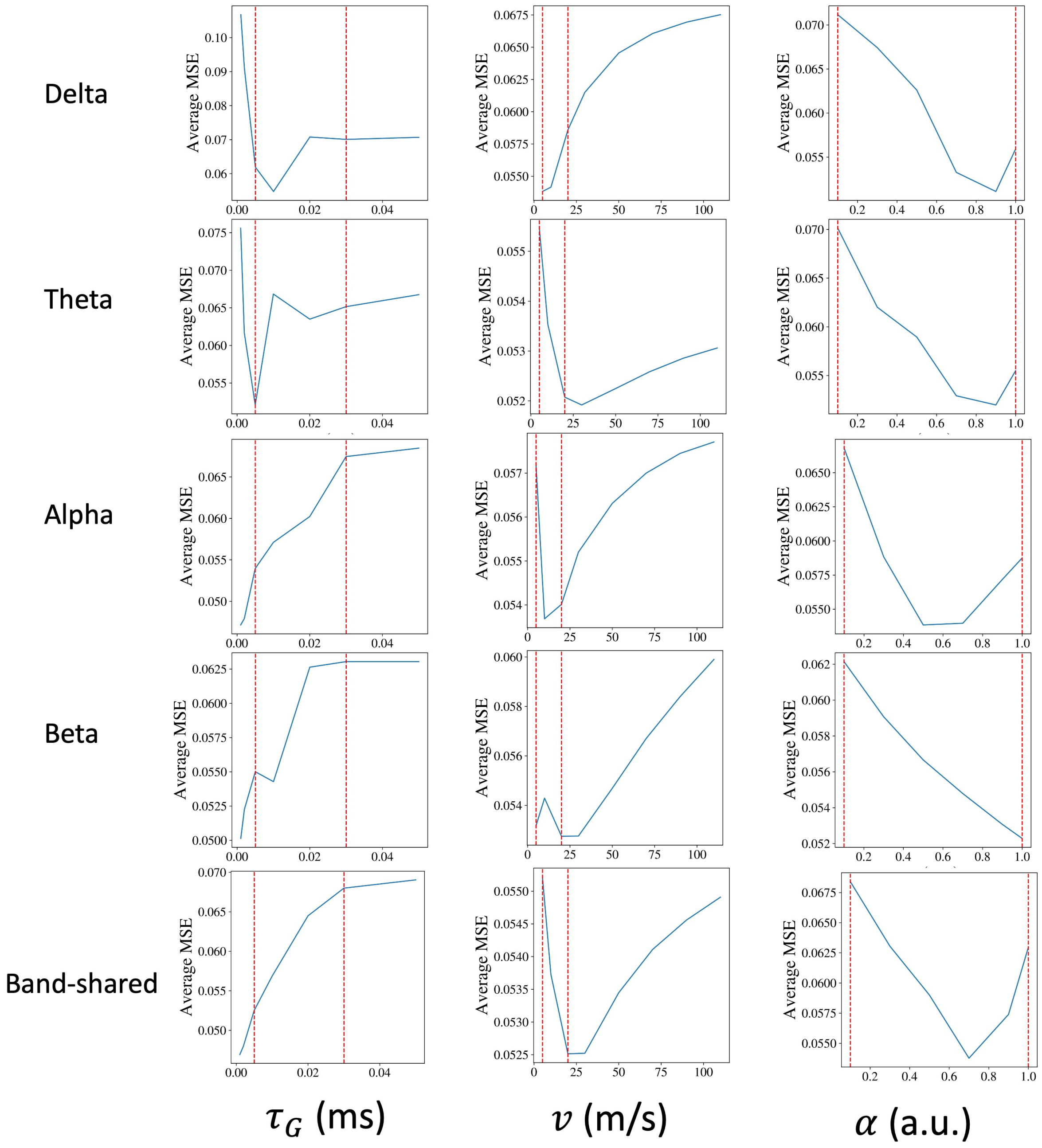

Figure S.4: Average MSE across 36 subjects between the empirical FC and model-predicted FC using the SGM-SBI model for different values of the SGM parameters. For each parameter, we vary it and fix the others to the optimal values obtained from the SBI algorithm. The red dashed lines represent the boundaries we used as indicated in Table 1.

References

(2020). *MATLAB version 9.8.0.1451342 (R2020a) Update 5*. The Mathworks, Inc., Natick, Massachusetts.

Abdelnour, F., Voss, H. U., and Raj, A. (2014). Network diffusion accurately models the relationship between structural and functional brain connectivity networks. *NeuroImage*, 90:335–347.

Dalal, S. S., Zumer, J., Agrawal, V., Hild, K., Sekihara, K., and Nagarajan, S. (2004). Nutmeg: a neuromagnetic source reconstruction toolbox. *Neurology & clinical neurophysiology: NCN*, 2004:52.

Fischl, B., Salat, D. H., Busa, E., Albert, M., Dieterich, M., Haselgrove, C., Kouwe, A. V. D., Killiany, R., Kennedy, D., Klaveness, S., Montillo, A., Makris, N., Rosen, B., and Dale, A. M. (2002). Whole Brain Segmentation : Automated Labeling of Neuroanatomical Structures in the Human Brain. *Neuron*, 33:341–355.

Jenkinson, M., Beckmann, C. F., Behrens, T. E., Woolrich, M. W., and Smith, S. M. (2012). FSL. *NeuroImage*, 62(2):782–790.

Owen, J. P., Li, Y.-O., Ziv, E., Strominger, Z., Gold, J., Bukhpun, P., Wakahiro, M., Friedman, E. J., Sherr, E. H., and Mukherjee, P. (2013). The structural connectome of the human brain in agenesis of the corpus callosum. *NeuroImage*, 70:340–355.
